# Roll with the punches: Fibroblast growth factor 10 alleviates pyroptosis of alveolar epithelial cells in different immune niches

**DOI:** 10.1002/ctm2.70569

**Published:** 2026-01-02

**Authors:** Tianchang Wei, Xiaoyan Chen, Jian Xu, Weiqi Mao, Zhenlin Yang, Yuhan Wang, Yufan Li, Wenting Jin, Cuicui Chen, Cuiping Zhang, Yuanlin Song

**Affiliations:** ^1^ Department of Pulmonary Medicine Shanghai Key Laboratory of Lung Inflammation and Injury Zhongshan Hospital, Fudan University Shanghai China; ^2^ Department of Pulmonary Medicine, Shanghai Institute of Infectious Disease and Biosecurity Shanghai China; ^3^ Department of Infectious Disease Zhongshan Hospital, Fudan University Shanghai China; ^4^ Department of Laboratory of Lung Inflammation and Injury Shanghai Respiratory Research Institute Shanghai China; ^5^ Department of National and Shanghai Clinical Research Center for Aging and Medicine Huashan Hospital, Fudan University Shanghai China; ^6^ Department of Key Laboratory of Chemical Injury, Emergency and Critical Medicine of Shanghai Municipal Health Commission, Center of Emergency and Critical Medicine Jinshan Hospital of Fudan University Shanghai China

**Keywords:** acute lung injury, acute respiratory distress syndrome, AMP‐activated protein kinase, fibroblast growth factor 10, pyroptosis

## Abstract

**Background:**

Acute respiratory distress syndrome (ARDS) is a life‐threatening condition characterized by high mortality with no specific treatments. Fibroblast growth factor 10 (FGF10) is recognized for its tissue repair and anti‐inflammatory roles in injured lungs; however, its clinical relevance and mechanistic role in ARDS remain unclear.

**Methods:**

Serum FGF10 levels were measured in patients with ARDS and analyzed for associations with clinical outcomes. An LPS‐induced mouse model of acute lung injury (ALI) was used to evaluate the effects of FGF10 treatment in vivo. Single‐cell RNA sequencing of lineage‐traced alveolar epithelial cells (AECs) was performed to identify transcriptional changes following FGF10 administration. In vitro co‐culture systems involving macrophages or neutrophils with AECs were established to investigate immune cell‐specific mechanisms.

**Results:**

We found that serum FGF10 levels were significantly reduced in ARDS patients, and this reduction correlated with poor prognosis. Moreover, FGF10 treatment alleviated lung inflammation by decreasing inflammatory cell infiltration and pro‐inflammatory cytokine release in mice. Leveraging single‐cell RNA sequencing of lineage tracing alveolar epithelial cells (AECs), we identified that the mRNA expression of *Ripk1*, *Casp8*, and *Casp3* were decreased after FGF10 treatment. In in vitro co‐culture experiments, we noticed that FGF10 did not inhibit macrophage pyroptosis. Instead, FGF10 effectively blocked the downstream RIPK1/caspase‐8/caspase‐3/gasdermin E (GSDME) signaling pathway in AECs. Additionally, FGF10 suppressed AMP‐activated protein kinase (AMPK) activation by modulating ATP production, thereby preventing RIPK1 cleavage.

**Conclusion:**

FGF10 alleviates acute lung injury by inhibiting AMPK‐RIPK1/caspase‐8/caspase‐3/GSDME‐mediated pyroptosis in AECs primed by distinct immune cell populations, supporting its potential as a therapeutic strategy for ARDS.

**Key points:**

- Our study reveals a marked decrease of serum FGF10 levels in ARDS patients, correlating with P/F ratio, hospitalisation days and mortality rates.
- We clarify how FGF10 prevents AECs' pyroptosis triggered by different immune cell infiltrations in different ways.
- FGF10 restored ATP levels to attenuate RIPK1 phosphorylation via AMPK to disrupt pyroptosis in the AECs.

## Graphical abstract

1. Our study reveals a marked decrease of serum FGF10 levels in ARDS patients, correlating with P/F ratio, hospitalisation days and mortality rates.
2. We clarify how FGF10 prevents AECs' pyroptosis triggered by different immune cell infiltrations in different ways.
3. FGF10 restored ATP levels to attenuate RIPK1 phosphorylation via AMPK to disrupt pyroptosis in the AECs.

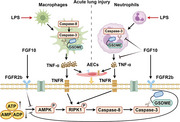

## 1 INTRODUCTION

Acute respiratory distress syndrome (ARDS) is a life‐threatening condition characterised by severe lung inflammation and diffuse alveolar injury, leading to impaired gas exchange and respiratory failure.^1^ Despite advances in critical care, the mortality rate of ARDS remains high, up to 40%, and no specific pharmacological treatments are currently available.^2^, ^3^ The pathophysiology of ARDS involves a complex interplay of inflammatory responses, oxidative stress and various forms of regulated cell death (RCD), including pyroptosis, apoptosis and necroptosis.^4^ Among these, pyroptosis – a highly inflammatory form of programmed cell death – has gained increasing attention due to its non‐negligible role in amplifying lung injury and promoting systemic inflammation.^5^

Pyroptosis is mediated by the activating gasdermin proteins, which are cleaved by caspases such as caspase‐1, caspase‐11 and caspase‐3. This process results in the formation of membrane pores, leading to cell lysis and the release of pro‐inflammatory cytokines, such as interleukin‐1β (IL‐1β) and IL‐18.^5^ In contrast to apoptosis, pyroptosis is characterised by its ability to elicit a robust inflammatory response, thereby amplifying an inflammatory cascade that causes tissue injury.^6^ Research indicates that pyroptosis of alveolar epithelial cells (AEC) is a crucial mechanism contributing to diffuse alveolar damage in ARDS.^7^, ^8^ AEC pyroptosis might also promote the formation of a cytokine storm, which can further worsen lung injury.^9^ Thus, targeting pyroptosis and its upstream signalling pathways offers a promising approach for mitigating lung injury and inflammation in ARDS patients.

Fibroblast growth factor 10 (FGF10), a member of the FGF family, is well known for its roles in embryonic lung development and alveolar regeneration after lung injury.^10^ FGF10 has been shown to maintain epithelial integrity by promoting the proliferation and survival of lung epithelial cells, which contributes to tissue repair and functional recovery.^11^, ^12^ Recent studies have expanded the functions of FGF10, highlighting its potential as an anti‐inflammatory and cytoprotective agent.^13^ FGF10 ameliorates the neurodegeneration of Alzheimer's disease by reducing neuronal apoptosis^14^ and protects hepatocytes and renal tubular epithelia from apoptosis in ischaemia–reperfusion injury.^15^, ^16^ Many molecular‐level pathways overlap between apoptosis and pyroptosis.^17^ Given the critical role of pyroptosis in ARDS pathogenesis,^5^ our study aims to investigate whether FGF10 attenuates lung inflammation and injury by inhibiting pyroptosis in AECs.

In this study, we found that FGF10 levels are significantly lower in ARDS patients, which correlates with poor prognosis. In the acute lung injury (ALI) mice model, we demonstrated that FGF10 treatment could effectively attenuate lung inflammation and injury. Furthermore, we identified that FGF10 mitigates ALI via inhibiting AMP‐activated protein kinase (AMPK)–RIPK1/caspase‐8/caspase‐3/GSDME‐mediated pyroptosis in AECs primed by different immune cells. By clarifying the protective effects and mechanisms of FGF10 in ALI, this research could facilitate the development of FGF10 for ARDS.

## 2 RESULT

### 2.1 FGF10 is reduced in ARDS patients and correlates with poor prognosis

**FIGURE 1:**
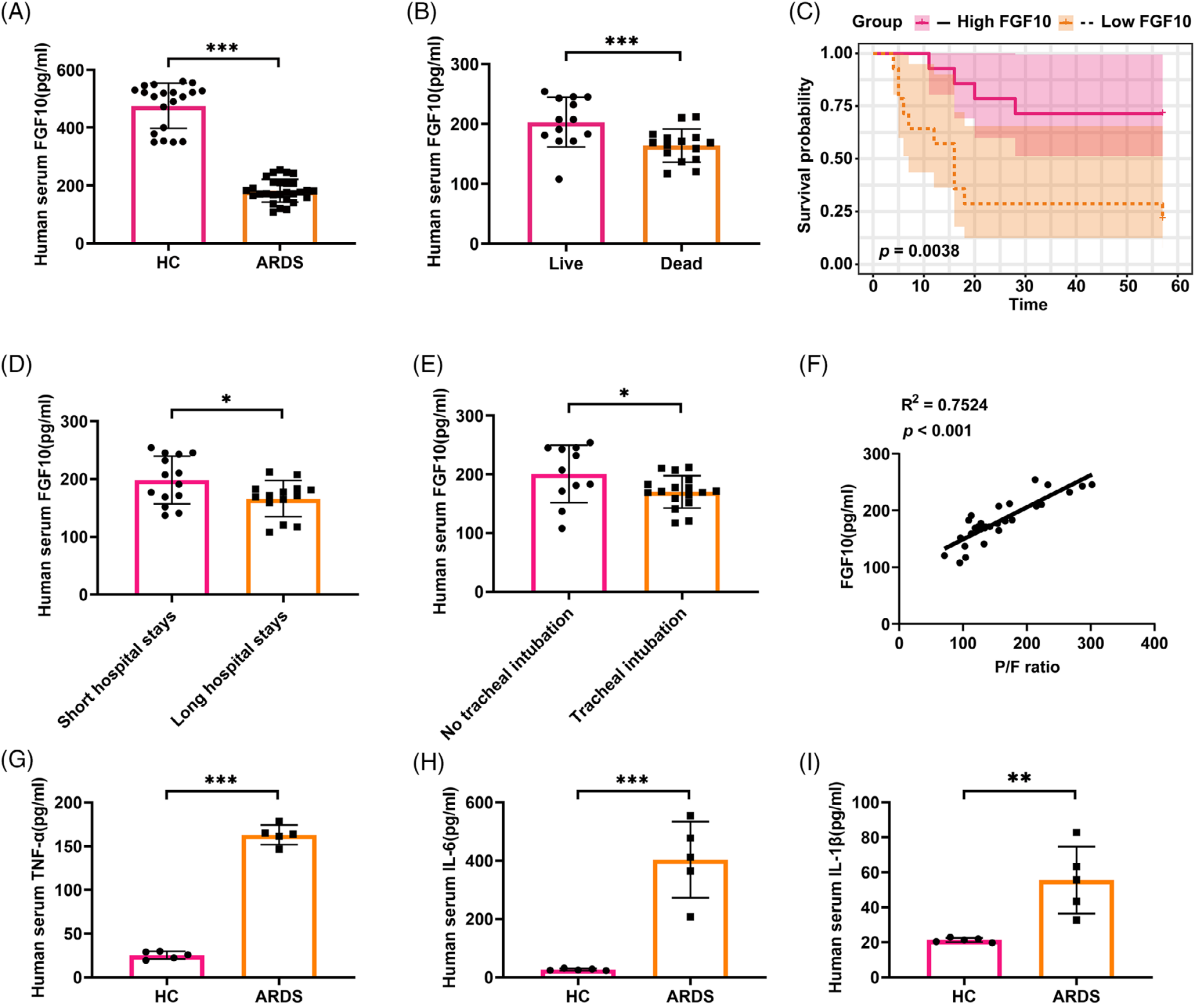
Serum FGF10 is decreased in ARDS patients and correlated with clinicopathological features. (A) Serum FGF10 levels of ARDS patients (*n* = 28) and healthy controls (*n* = 20) measured by ELISA. (B) The serum FGF10 levels in the survival group relative to the non‐survival group of ARDS patients. (C) The Kaplan–Meier survival analysis of ARDS patients between the high‐FGF10 group and the low‐FGF10 group (stratified by median serum FGF10 levels, log‐rank *p* = .0038). (D) The serum FGF10 levels of ARDS patients in long hospital stays relative to short hospital stays (classified by median days of hospitalisation). (E) The serum FGF10 levels in patients requiring tracheal intubation relative to non‐intubation. (F) The correlation analysis between serum FGF10 levels and P/F ratios (*R* ^2^ = .72, *p *< .001). (G–I) Serum levels of TNF‐α, IL‐6 and IL‐1β were quantified by ELISA (*n* = 5). Statistical significance was determined by a two‐sided *t*‐test. Data are shown as mean ± SD (*p < .05, **p < .01, ***p < .001).

To investigate the role of FGF10 in the development of ARDS, we collected serum samples from 28 ARDS patients and 20 healthy controls (patient characteristics are detailed in Table 1). We measured serum FGF10 levels in these individuals and found that FGF10 levels were significantly lower in the ARDS patients compared with the healthy controls (*p* < .001), as shown in Figure 1A. Further stratification of ARDS patients based on their prognosis showed that serum FGF10 levels were significantly higher in the survival group compared with the non‐survival group (*p* < .001) (Figure 1B). The Kaplan–Meier survival curves indicated that patients in the high‐FGF10 group had significantly more favourable overall survival compared with patients in the low‐FGF10 group during the follow‐up period (*p* < .01) (Figure 1C). Additionally, patients with long hospital stays and those requiring tracheal intubation exhibited significantly lower serum levels of FGF10 (Figure 1D,E). Pearson correlation analysis revealed a positive correlation between serum FGF10 levels and the PaO_2_/FiO_2_ (P/F) ratio (*R* ^2^ = .7524, *p* < .001), suggesting a potential relationship between reduced FGF10 levels and hypoxia (Figure 1F). Additionally, we measured the levels of pro‐inflammatory cytokines, TNF‐α, IL‐6 and IL‐1β in the serum. The serum concentration of pro‐inflammatory cytokines was significantly elevated in ARDS patients compared with healthy controls (Figure 1G–I). Collectively, these findings suggest that decreased serum FGF10 levels were associated with disease severity, prolonged hospitalisation and a poor prognosis in ARDS patients.

**TABLE 1:** Baseline characteristics of healthy controls (HC) and ARDS patients.

	ARDS (*n* = 28)	Healthy control (*n* = 20)	*p* value
Gender (*n*, %)			.545
Male	9 (32.1%)	9 (45.0%)	
Female	19 (67.9%)	11 (55.0%)	
Ages (years)	63.5 (16.4)	64.4 (14.1)	.846
BMI (kg/m^2^)	23.7 (4.04)	23.4 (2.46)	.735
White blood cells (10^9^/L)	13.7 (7.41)	5.46 (1.52)	<.001
Neutrophils (%)	88.9 (5.99)	58.3 (10.5)	<.001
Lymphocytes (%)	5.31 (3.21)	29.2 (5.43)	<.001
Monocytes (%)	4.86 (3.35)	7.66 (1.80)	<.01
Platelets (10^9^/L)	173 (92.2)	175 (76.9)	.944
CRP (mg/L)	81.8 (15.9)	12.7 (17.2)	<.001
LDH (U/L)	453 (399)	175 (28.8)	<.01

### 2.2 FGF10 mitigates LPS‐induced ALI

To evaluate the role of FGF10 in a mouse model, we assessed FGF10 levels in serum 24 h after LPS administration via intratracheal (i.t.) injection in mice. We observed a significant decrease in serum FGF10 levels in the LPS‐treated group compared with the control group (Figure S1A). In contrast, pro‐inflammatory cytokines, TNF‐α, IL‐6 and IL‐1β, were significantly elevated in the serum of LPS‐treated mice (Figure S1B–D). Concurrently, we examined the expression of FGF10 and its receptor, FGFR2b, in the lung tissue through western blot and quantitative PCR (qPCR). Negligible staining for FGF10 and FGFR2b was observed in lungs from the LPS group (Figure S1E–G). Correspondingly, the mRNA expression levels of *Fgf10* and *Fgfr2b* were significantly lower in the LPS group than in the control group (Figure S1H,I).

**FIGURE 2:**
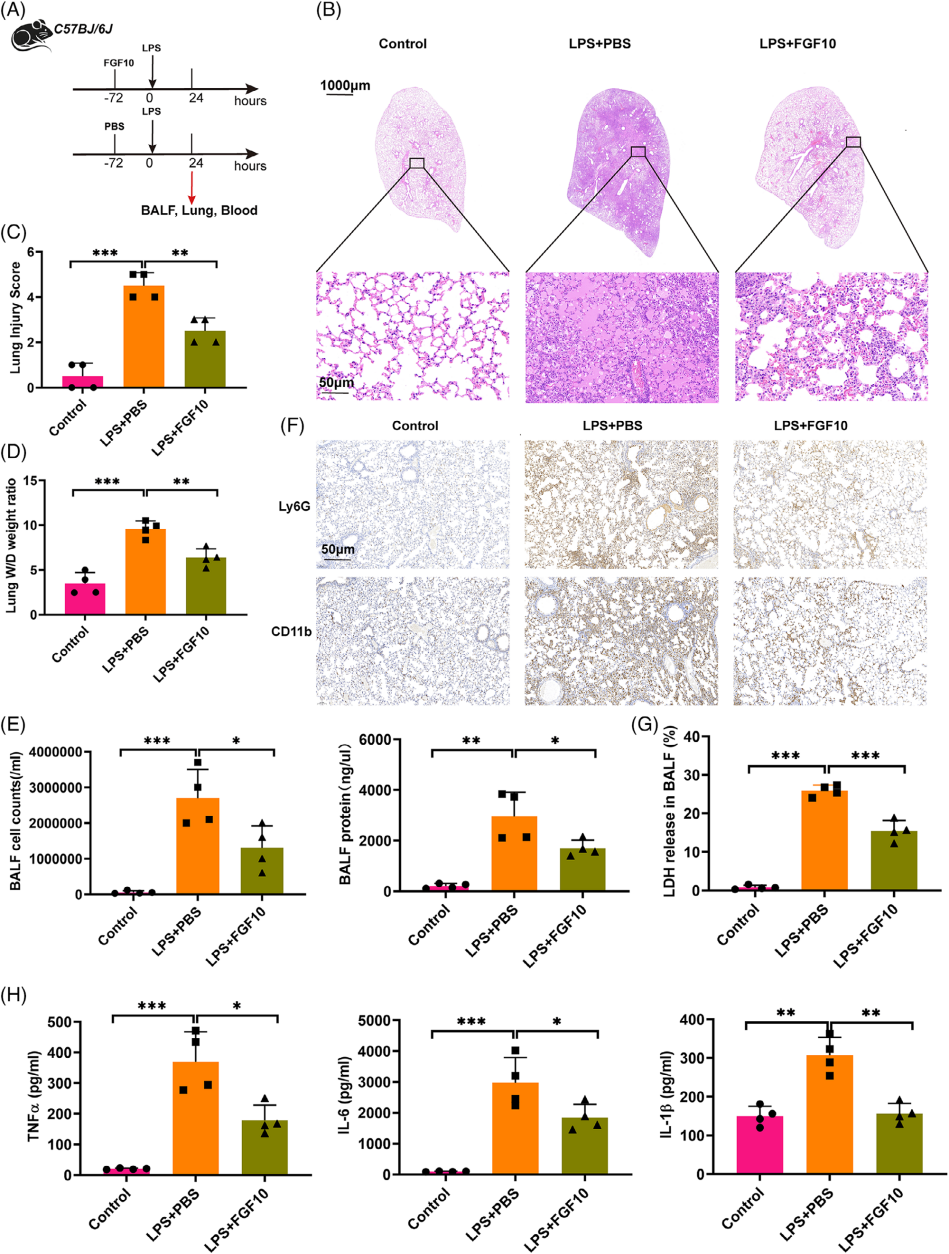
FGF10 attenuates LPS‐induced ALI in mice. (A) Schematic representation of experimental design: *C57BL/6* mice received intratracheal injection of FGF10 (5 mg/kg) or PBS 72 h prior to LPS challenge (5 mg/kg, intratracheal). Lung tissues, blood and BALF were harvested 24 h after LPS administration. (B) Representative H&E‐stained lung sections (scale bar: 1000 and 50 µm). (C) Quantitative lung injury scores (0–4 scale) based on H&E morphology. (D) Lung W/D weight ratio, indicating pulmonary oedema. (E) Total cell counts (left) and protein concentration (right) were analysed in BALF. (F) Immunohistochemical staining for Ly6G and CD11b in lung tissues (scale bar: 50 µm). (G) Detection of LDH level in BALF. (H) Levels of pro‐inflammatory cytokines (TNF‐α, IL‐6, IL‐1β) in BALF measured by ELISA. Statistical significance was determined by one‐way ANOVA with Tukey's post hoc test. Four independent experiments are carried out. Data are shown as mean ± SD (*n* = 4, *p < .05, **p < .01, ***p < .001).

To explore the potential role of FGF10 in mitigating lung injury, we intratracheally treated mice with FGF10 72 h before LPS exposure (Figure 2A). Histological analysis of lung tissues showed that FGF10 treatment reduced immune cell infiltration, alleviated pulmonary oedema and resulted in thinner alveolar‐capillary walls compared with the PBS–LPS group (Figure 2B). Compared with control mice, the lung injury score of the FGF10‐treated mice was significantly lower (Figure 2C). The wet‐to‐dry weight ratio of lung tissue was significantly reduced in the FGF10‐treated mice, suggesting that FGF10 treatment helped to maintain the integrity of the air–vascular barrier and decreased pulmonary oedema (Figure 2D).

To further explore the anti‐inflammatory role of FGF10, we evaluated the levels of inflammatory markers in bronchoalveolar lavage fluid (BALF). We found that both the cell counts and total protein concentration in the BALF were significantly decreased in the FGF10–LPS group compared with the PBS–LPS group, suggesting a reduction in alveolar inflammation (Figure 2E). Additionally, FGF10 treatment led to a decreased infiltration of neutrophils in lung tissue (Figure 2F). Levels of LDH release and pro‐inflammatory cytokines, such as TNF‐α, IL‐6 and IL‐1β, were significantly lower in lung tissue and BALF in the FGF10–LPS group compared with the PBS–LPS group (Figure 2G,H). These findings suggest that FGF10 effectively alleviates lung inflammation and tissue injury.

### 2.3 FGF10 inhibits LPS‐induced *Ripk1*, *Casp8* and *Casp3* expression in the AECs in vivo

**FIGURE 3:**
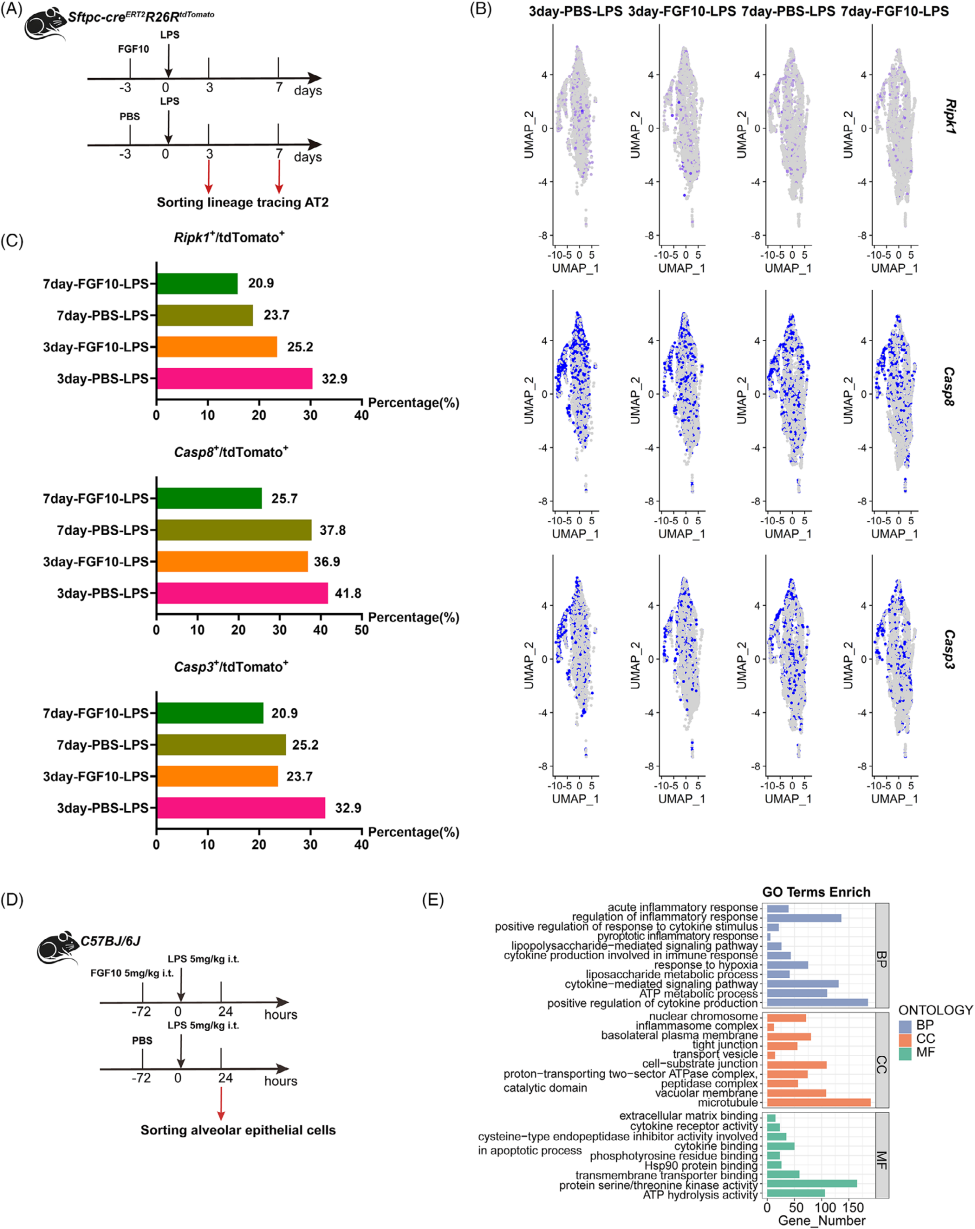
FGF10 suppressed *Ripk1*, *Casp8* and *Casp3* in alveolar epithelial cells. (A) Schematic representation of experimental design: Sftpc‐cre^ERT2^R26R^tdTomato^ lineage‐tracing mice were pretreated intratracheally with FGF10 (5 mg/kg) or PBS 72 h before LPS challenge (2.5 mg/kg). Lineage‐tracing AT2 cells were isolated for scRNA‐seq at days 3 and 7 post‐LPS. (B) UMAP projection of scRNA‐seq data from AT2 cells, coloured by expression levels of *Ripk1*, *Casp8* and *Casp3*. (C) Quantification of *Ripk1^+^*, *Casp8^+^* and *Casp3^+^* AT2 cell proportions. (D) RNA‐seq experimental design: *C57BL/6* mice received FGF10/PBS pretreatment as in (A) and LPS (5 mg/kg). AECs were sorted 24 h post‐LPS for transcriptomic profiling (*n* = 4). (E) GO enrichment analysis of DEGs in PBS+LPS versus FGF10+LPS groups. ScRNA‐seq processed using Seurat v4.0; DEGs filtered by |log_2_FC| > 1 and FDR < .05.

The extensive death of AECs leads to diffuse lung injury in ARDS.^18^ Current studies suggest that activation of RCD pathways, mainly including apoptosis,^19^ pyroptosis^9^, ^17^ and necroptosis,^20^ significantly contributes to the destruction of AEC during lung injury. To verify whether FGF10 attenuates lung injury by interfering with the RCD pathway of AEC, we performed single‐cell RNA sequencing (scRNA‐seq) of lineage tracing AECs isolated from PBS–LPS or FGF10–LPS treated mice at 3 and 7 days (Figures 3A and S2). Notably, we found that the mRNA expression of *Xiap*, *Bax, Ripk1, Casp8* and *Casp3* in the lineage‐labelled AECs was significantly reduced after FGF10 treatment compared with PBS‐treated controls (Figure 3B,C), suggesting that FGF10 may attenuate lung injury by regulating apoptosis or pyroptosis in ALI. Previous studies have shown that FGF10 plays a role in regulating apoptosis.^21^, ^22^ However, we believe that pyroptosis – a form of programmed necrosis characterised by the release of various inflammatory mediators and cellular debris – plays a more significant role in the excessive inflammatory damage associated with ARDS.^23^

To further validate our findings, we sorted out the AECs 24 h after LPS administration before performing transcriptome sequencing (RNA‐seq) (Figure 3D). Gene Ontology (GO) enrichment analysis, based on the differentially expressed genes (DEGs) between the PBS–LPS group and FGF10–LPS group, revealed that the FGF10–LPS group was significantly enriched in biological processes related to pyroptotic inflammatory response and cytokine‐mediated signalling pathways (Figure 3E).

### 2.4 FGF10 suppresses pyroptosis of the AECs in ALI mice

**FIGURE 4:**
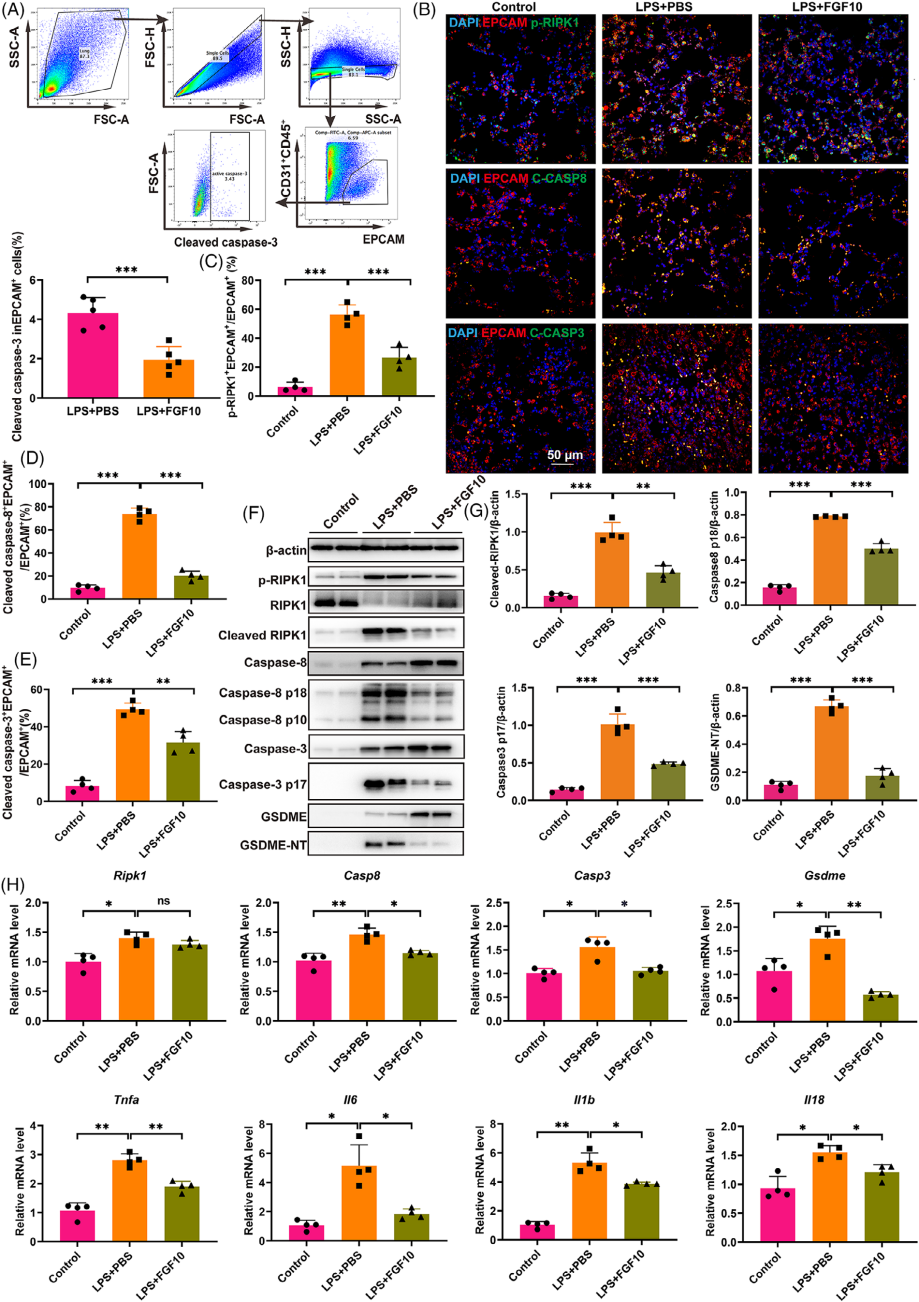
FGF10 inhibits GSDME‐dependent pyroptosis in alveolar epithelial cells. (A) Flow cytometry quantification of cleaved caspase‐3⁺ cells within EPCAM⁺ alveolar epithelial populations. (B) Representative immunofluorescence staining of p‐RIPK1 (Ser166), cleaved caspase‐8 and cleaved caspase‐3 (green) in EPCAM⁺ cells (red) with DAPI nuclear counterstain (blue) (scale bar: 50 µm). (C–E) Quantitative analysis of p‐RIPK1⁺ (C), caspase‐8⁺ (D) and caspase‐3⁺ (E) cells in EPCAM⁺ populations. (F) Western blot analysis of RIPK1, caspase‐8, caspase‐3 and GSDME in sorted alveolar epithelial cells. (G) Statistical analysis of protein activation of RIPK1, caspase‐8, caspase‐3 and GSDME (normalised to β‐actin). (H) qRT‐PCR analysis of *Ripk1, Casp8, Casp3, Gsdme* and pro‐inflammatory cytokines (*Tnfa*, *Il6*, *Il1b*, *Il18*) mRNA levels. Statistical significance was determined by a two‐sided *t*‐test or one‐way ANOVA with Tukey's post hoc test. Four independent experiments are carried out Data are shown as mean ± SD (*n* = 4, *p < .05, **p < .01, ***p < .001).

To further investigate how FGF10 regulates pyroptosis, we examined the activation of caspase‐3 in AECs of the ALI mice using flow cytometry and the activation of caspase‐3, caspase‐8 and RIPK1 using immunofluorescence staining. The results showed that cleavage of caspase‐8 and caspase‐3 and phosphorylation of RIPK1 in the AECs were significantly increased in the LPS‐treated mice compared with the PBS control. However, this increase was significantly reversed by FGF10 treatment (Figure 4A–E). This finding indicates that FGF10 might inhibit both the expression and activation of RIPK1, caspase‐3 and caspase‐8 in AEC.

In addition, we isolated AECs from the PBS–LPS and FGF10–LPS groups and confirmed that LPS activated the pyroptosis pathway in AECs, as evidenced by the cleavage of GSDME into its N‐terminal fragment (GSDME‐NT). And FGF10 intervention effectively inhibited GSDME‐mediated pyroptosis (Figure 4F,G). The qPCR analyses of related genes were consistent with the protein expression (Figure 4H). These results further suggest that FGF10 might alleviate ALI by suppressing pyroptosis in AECs.

### 2.5 FGF10 prevents AEC pyroptosis induced by LPS‐primed macrophages

**FIGURE 5:**
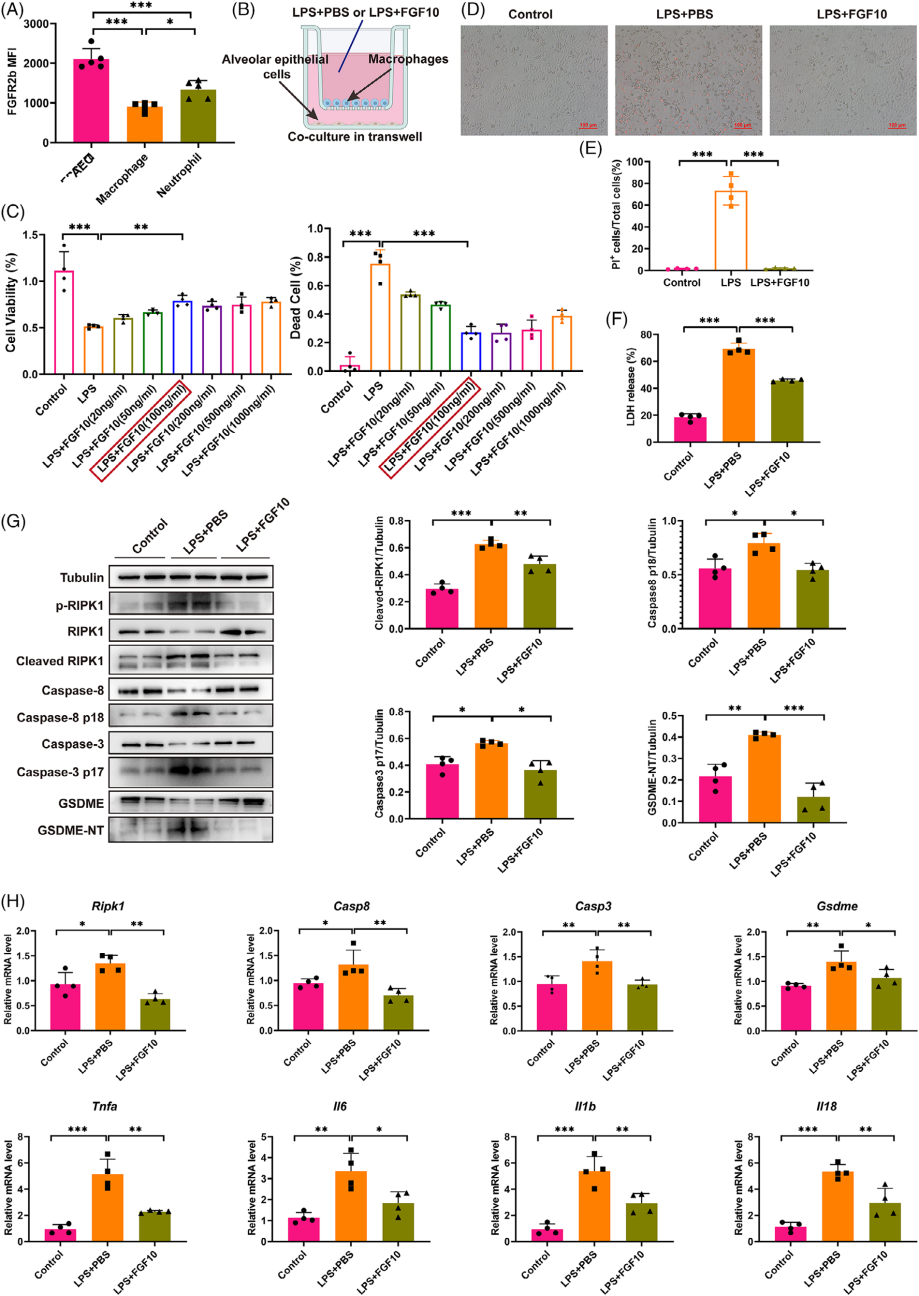
FGF10 inhibits MLE12 pyroptosis induced by LPS‐primed iBMDMs. (A) Flow cytometry quantification of FGFR2b⁺ cells in alveolar epithelial cells (EPCAM⁺), macrophages (CD11b⁺F4/80⁺) and neutrophils (CD11b⁺Ly6G⁺). (B) Schematic representation of MLE12 and iBMDMs co‐culture system. Cells were pre‐treated with PBS/FGF10 (100 ng/mL) for 6 h and then interfered with LPS (1 µg/mL) for 6 h. (C) Cell viability assessed by CCK‐8 assay (left) and PI⁺ dead cell proportion (right). (D) Representative PI staining images (red: dead cells, scale bar: 100 µm). (E) Quantification of PI⁺ MLE12 cells. (F) Detection of LDH level in MLE12 cells. (G) Western blot analysis of RIPK1, caspase‐8, caspase‐3 and GSDME in co‐cultured MLE12 cells. Statistical analysis of protein activation of RIPK1, caspase‐8, caspase‐3 and GSDME (normalised to tubulin). (H) qRT‐PCR analysis of *Ripk1, Casp8, Casp3, Gsdme* and pro‐inflammatory cytokines (*Tnfa*, *Il6*, *Il1b*, *Il18*) in MLE12 cells. Statistical significance was determined by one‐way ANOVA with Tukey's post hoc test. Four independent experiments are carried out. Data are shown as mean ± SD (*n* = 4, *p < .05, **p < .01, ***p < .001).

Consistent with our previous study,^24^ LPS did not directly cause RIPK1/caspase‐3/caspase‐8/GSDME‐induced pyroptosis in AEC but might induce AEC pyroptosis by inflammatory cells (Figure S3A,B). To explore how FGF10 intervenes with AEC pyroptosis, we assessed the expression of the FGF10 receptor, FGFR2b, on AEC, macrophages and neutrophils. In accordance with the previous study,^25^ FGF2Rb was predominantly expressed in AECs (Figures 5A and S3C,D). We then co‐cultured MLE12 with iBMDMs and pre‐treated them with a range of concentrations of FGF10 prior to LPS stimulation (Figure 5B). We observed that FGF10 significantly increased cell viability and decreased the proportion of dead cells after LPS challenge at the concentration of 100 ng/mL (Figure 5C), which would be employed for the subsequent experiments. Propidium iodide (PI) uptake assay verified that FGF10 reduced the necrosis rate of MLE12 (Figure 5D,E). Levels of LDH release were significantly lower in MLE12 cells in the FGF10–LPS group compared with the PBS–LPS group (Figure 5F). Western blotting confirmed the activation of GSDME‐induced pyroptosis signalling cascade measured by the phosphorylation of RIPK1 and the cleavage of caspase‐8, caspase‐3 and GSDME in MLE12, which co‐cultured with iBMDMs under LPS challenge. And FGF10 could inhibit the pyroptotic signal cascade (Figure 5G). FGF10 also reduced the mRNA expression of *Ripk1*, *Casp8*, *Casp3* and *Gsdme* in MLE12, consistent with the in vivo experiments (Figure 5H).

Meanwhile, we found that FGF10 also suppressed the mRNA expression of pro‐inflammatory cytokines, *Tnfa*, *Il6*, *Il1b* and *Il18*, of MLE12 (Figure 5H). But when we evaluated the release of the pro‐inflammatory factors TNF‐α, IL‐1β and IL‐6 in the transwell culture medium, we found that pre‐treatment with FGF10 significantly inhibited the release of IL‐1β and IL‐6 but not the release of TNF‐α (Figure S3E). Therefore, during the macrophage‐dominated immunological response, FGF10 can inhibit macrophage‐induced AEC pyroptosis and reduce production of proinflammatory cytokines.

### 2.6 FGF10 inhibits neutrophil pyroptosis and prevents AEC pyroptosis induced by LPS‐primed neutrophils

**FIGURE 6:**
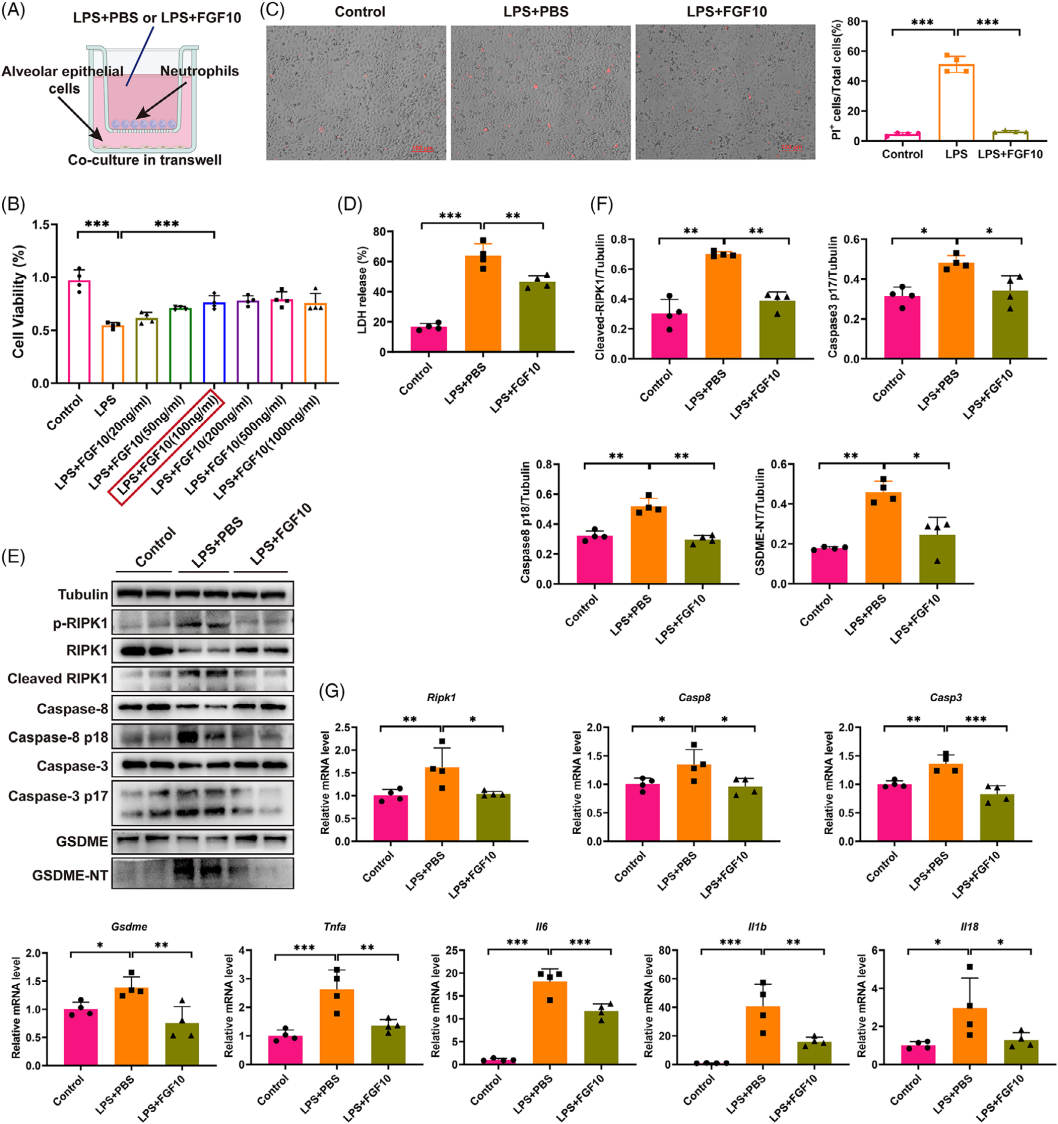
FGF10 inhibits MLE12 pyroptosis induced by LPS‐primed neutrophils. (A) Schematic representation of MLE12‐neutrophil co‐culture system. Primary murine neutrophils were co‐cultured with MLE12 cells and treated with FGF10 (100 ng/mL) for 6 h and then LPS (1 µg/mL) for 6 h. (B) CCK‐8 assay showed MLE12 viability. (C) PI staining images (red: dead cells, scale bar: 100 µm) and quantification of PI⁺ MLE12 cells. (D) Detection of LDH release in MLE12 cells. (E) Western blot analysis of RIPK1, caspase‐8, caspase‐3 and GSDME in MLE12 cells. (F) Densitometric quantification of protein activation of RIPK1, caspase‐8, caspase‐3 and GSDME (normalised to tubulin). (G) mRNA levels of *Ripk1*, *Casp8*, *Casp3*, *Gsdme* and pro‐inflammatory cytokines (*Tnfa*, *Il6*, *Il1b*, *Il18*) in co‐cultured MLE12 cells are evaluated by qRT‐PCR. Statistical significance was determined by one‐way ANOVA with Tukey's post hoc test. Four independent experiments are carried out. Data are shown as mean ± SD (*n* = 4, *p < .05, **p < .01, ***p < .001).

As we all know, neutrophils are also the main drivers of the early acute lung inflammatory response in patients with ARDS.^26^ It has also been shown that neutrophils not only undergo pyroptosis but that the neutrophil extracellular traps they produce also induce pyroptosis in surrounding cells.^27^ Using the same method as above, we co‐cultured neutrophils with MLE12 cells and treated them with LPS and FGF10 (Figure 6A). The cell viability assays revealed that FGF10 at 100 ng/mL significantly improved the MLE12 cell viability after LPS challenge (Figure 6B). PI staining and LDH release validated that LPS stimulation induced significant pyroptosis of MLE12 cells co‐cultured with neutrophils and FGF10 markedly reduced MLE12 pyroptosis rate (Figure 6C,D). Western blotting verified that neutrophils also induced GSDME‐mediated pyroptosis in MLE12 under LPS challenge, which was confirmed by the activation of the RIPK1/caspase‐8/caspase‐3/GSDME pyroptotic signal pathway (Figure 6E,F). FGF10 pre‐treatment also inhibited the activation and mRNA expression of this pyroptotic signal pathway activated by LPS‐primed neutrophil (Figure 6G). The findings affirmed the protective effect of FGF10 against pyroptosis in AECs.

Previous research showed that neutrophil pyroptosis occurred via the PR3/caspase‐3/GSDME pathway.^28^ Interestingly, we observed that LPS activated caspase‐3 and GSDME in neutrophils, which was significantly reversed by FGF10 (Figure S4A,B). The expression and release of the pro‐inflammatory cytokines, such as TNF‐α, IL‐6, IL‐1β and IL‐18, of neutrophils were also reduced by FGF10 (Figure S4C). Therefore, during the neutrophil‐infiltration‐dominated ALI phase, FGF10 can inhibit neutrophil pyroptosis and reduce TNF‐α release, as well as prevent AEC pyroptosis.

### 2.7 FGF10 prevents AEC pyroptosis by suppressing RIPK1 via ATP/AMPK pathway

**FIGURE 7:**
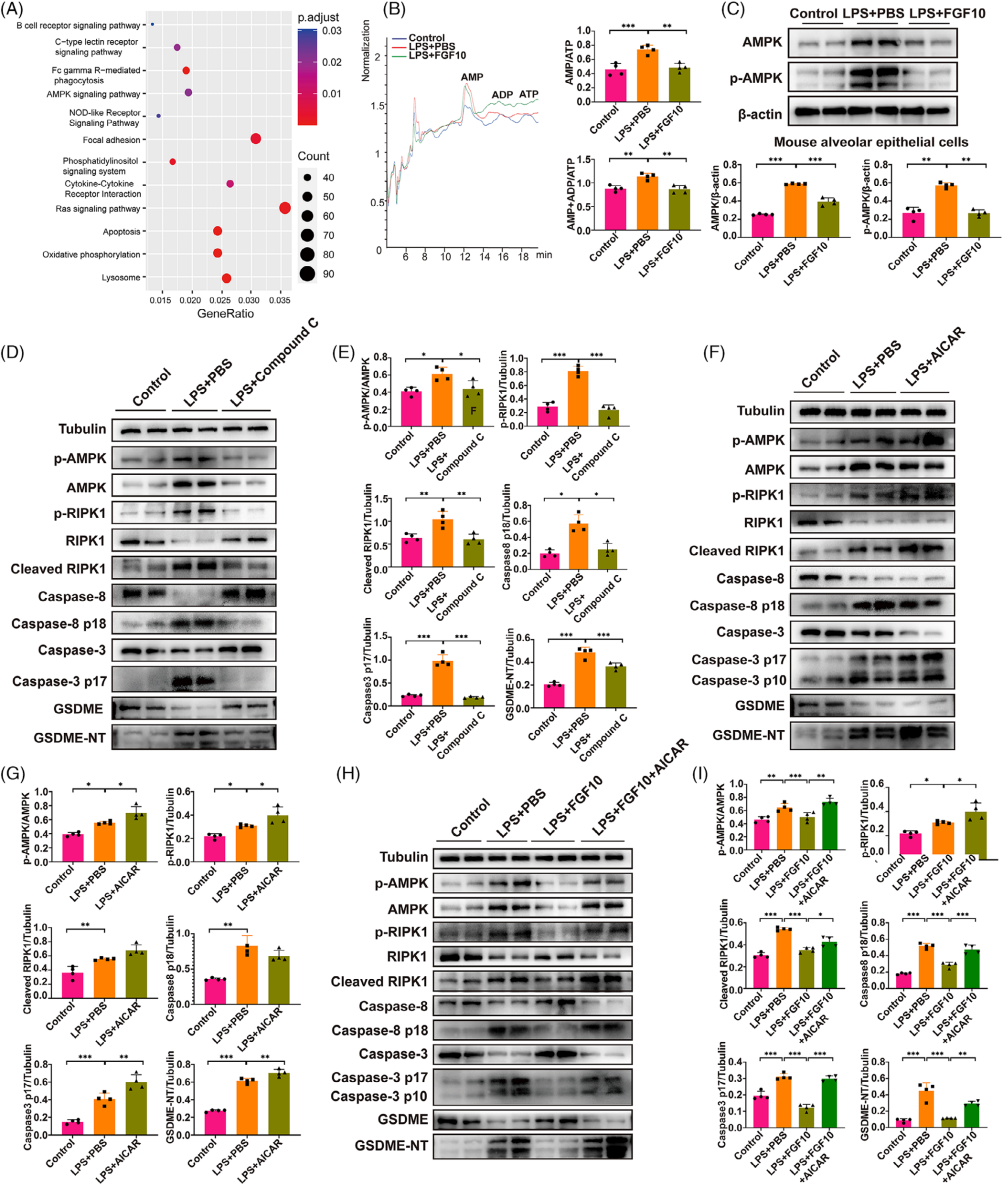
FGF10 inhibits AMPK to suppress RIPK1/caspase‐8/caspase‐3/GSDME axis. (A) KEGG pathway enrichment analysis of RNA‐seq data from PBS+LPS versus FGF10+LPS groups. (B) HPLC quantification of AMP, ADP and ATP in MLE12 cells. (C) Western blot and statistical analysis of phospho‐AMPKα (Ser166) and total AMPKα in alveolar epithelial cells. (D–I) Pharmacological modulation of AMPK in co‐cultured MLE12 cells showed by western blot and statistical analysis: D and E: Compound C (5 µM), F and G: AICAR (1 mM), H and I: FGF10 and AICAR. Statistical significance was determined by one‐way ANOVA with Tukey's post hoc test. Four independent experiments are carried out. Data are shown as mean ± SD (*n* = 4, *p < .05, **p < .01, ***p < .001).

Next, we further investigate the detailed molecular mechanism of how FGF10 inhibits AEC pyroptosis. Our RNA sequencing analysis of AEC revealed FGF10 modulated ATP metabolism and the AMPK pathway (Figure 7A), while AMPK has been shown to activate GSDME‐mediated pyroptosis before.^29^ Previous studies reported that an increased AMP/ATP and AMP+ADP/ATP ratio triggers AMPK phosphorylation.^30^ Consistently, our results showed that LPS increased of the AMP/ATP ratio and (AMP+ADP)/ATP ratio (Figure 7B), which was subsequently remarkably suppressed by FGF10 treatment and subsequently inhibited AMPK phosphorylation in MLE12 cells co‐cultured with or without macrophages or neutrophils (Figure S5A–C). PI3K–AKT may be the main regulator of FGF10 on AMPK, which may be achieved by AKT‐dependent regulation of cellular energy metabolism (Figure S5D,E).^31^, ^32^ Likewise, FGF10 impaired LPS‐induced phosphorylation of AMPK in AECs isolated from ALI mouse lung treated with PBS or FGF10 (Figure 7C). As reported before,^33^ AMPK phosphorylation caused RIPK1 phosphorylation at Ser^415^ to suppress RIPK1. In our study, we focused on the relationship between AMPK and phosphorylation of RIPK1 at Ser^166^. Our results showed that phosphorylation of AMPK can induce phosphorylation of RIPK1 at Ser^166^, thereby activating RIPK1/caspase‐8/caspase‐3/GSDME‐mediated pyroptosis (Figures 4E, 5F, 6D and S5A,B). The immunofluorescence staining results demonstrated that FGF10 treatment significantly reduced LPS‐induced RIPK1 phosphorylation in MLE12 cells, which was considered to activate the caspase‐8/caspase‐3/GSMDE pyroptosis signalling pathway (Figure 4B–C,E). Collectively, these findings lend support to the hypothesis that FGF10 alleviates AEC pyroptosis via the ATP/AMPK–RIPK1 signalling pathway.

To verify our hypothesis, further experiments were performed using both an AMPK inhibitor (Compound C) and an activator (AICAR). We found that Compound C treatment reduced RIPK1 phosphorylation and inhibited caspase‐8/caspase‐3/GSMDE‐mediated pyroptosis, whereas activation of AMPK by AICAR had an opposite effect, significantly increasing RIPK1 phosphorylation and caspase‐8/caspase‐3/GSMDE‐mediated pyroptosis (Figure 7D–G). Furthermore, rescue experiments were conducted, where the effects of FGF10 on RIPK1 phosphorylation and pyroptosis were reversed by the AICAR (Figure 7H,I). Taken together, these results demonstrate that FGF10 inhibits AMPK activation, which subsequently reduces RIPK1 phosphorylation, thus protecting AEC from pyroptosis.

## 3 MATERIAL AND METHODS

### 3.1 Mice and ALI model

*C57BL/6J* mice (6–8 weeks old), including male and female animals, were purchased from Shanghai JieSiJie Laboratory Animal Co. Ltd. (Shanghai, China). *Sftpc‐cre^ERT2^ (B6.129S‐Sftpc^tm1(cre/ERT2)Blh^/J)* mice were generously provided by the Laboratory Chronic Airways Diseases, Department of Respiratory and Critical Care Medicine, Nanfang Hospital, Southern Medical University (Guangzhou, China). *Ai9 (B6.Cg‐Gt(ROSA)26Sor^tm9(CAG‐tdTomato)Hze^/J)* mice were kindly donated by Dr Zilong Qiu (the State Key Laboratory of Neuroscience, Center for Excellence in Brain Science and Intelligence Technology, Chinese Academy of Sciences.). We generated *Sftpc‐cre^ERT2^R26R^tdTomato^* mice by crossing *Sftpc‐cre^ERT2^* mice with Ai9 mice. Mice were housed in a pathogen‐free environment with a 12‐h light/dark cycle and unlimited access to food and water. All experimental procedures were approved by the Institutional Animal Care and Use Committee of Fudan University (Approval No. 202408FD0001) and conducted in compliance with the Guide for the Care and Use of Laboratory Animals (8th edition, 2011) published by the National Institutes of Health.

The mice were randomly divided into three groups: (1) control group (PBS–PBS), (2) LPS group (PBS–LPS) and (3) FGF10–LPS group. Before the experiment, mice were anesthetised via intraperitoneal injection of Avertin (Sigma–Aldrich; Cat. No. T48402). For the FGF10–LPS group, mice received an i.t. administration of FGF10 (5 mg kg^−1^; Peprotech; Cat. 100‐26‐25) 72 h before LPS challenge. For the PBS–PBS and PBS–LPS groups, mice received PBS (1 mL kg^−1^) instead. Afterward, mice in PBS–PBS groups were intratracheally delivered with PBS (1 mL kg^−1^), while mice in PBS–LPS and FGF10–LPS groups received LPS (5 mg kg^−1^; i.t.; Sigma–Aldrich; Cat: L9143) to induce ALI. *C57BL/6J* mice were euthanised on day 1, and *Sftpc‐cre^ERT2^R26R^tdTomato^* mice were euthanised on day 3 and day 7 using an overdose of Avertin.

### 3.2 Human participants

This study was approved by the Ethics Committee of Zhongshan Hospital, Fudan University (Approval No. B2021‐183R) and conducted in accordance with the principles outlined in the Declaration of Helsinki. Patients who presented to Zhongshan Hospital between July 2022 and June 2024 were enrolled in the study after providing written informed consent. The diagnosis of ARDS was made according to the 2023 updated definition of ARDS.^34^ The control group had no ARDS, infections, interstitial lung disease, asthma, COPD or connective tissue disease (Table 1).

### 3.3 Enzyme‐linked immunosorbent assay

Blood/BALF from human and mice and cell culture supernatants were collected and analysed for FGF10, TNF‐α, IL‐6 or IL‐1β, which were detected by enzyme‐linked immunosorbent assay (ELISA) kits, following the manufacturer's instructions. Human and mouse TNF‐α (Cat: #430204, Cat: #430904), IL‐1β (Cat: #437004, Cat: #432604) and IL‐6 (Cat: #430504, Cat: #431304) ELISA kits were obtained from BioLegend. Human and mouse FGF10 (Cat: Hm11485, Cat: Mu31457) ELISA kits were purchased from Bioswamp. Briefly, samples were added to pre‐coated 96‐well plates, and optical density was measured at the specified wavelength with a microplate reader. Concentrations were determined from standard curves and expressed as pg/mL.

### 3.4 Lung histology

Lung tissues were collected, fixed in 4% paraformaldehyde, embedded in paraffin and sectioned for histological analysis. Haematoxylin and eosin (H&E) staining was performed to evaluate the degree of lung injury. A blinded scoring system evaluated the severity of lung damage: 0 = no injury; 1 = injured area < 25%; 2 = injured area between 25 and 50%; 3 = injured area between 50 and 75%; 4 = injured area > 75%.^35^ Quantitative scoring was conducted based on the proportion of damaged lung tissue observed under light microscopy.

### 3.5 Immunohistochemistry

Lung tissues were fixed, paraffin‐embedded, sectioned and processed to antigen retrieval. After blocking, sections were incubated with primary antibodies Ly6G (Servicebio; Cat: GB11229) and CD11b (Servicebio; Cat: GB115689) overnight at 4°C, followed by HRP‐conjugated secondary antibodies and DAB staining. Nuclei were counterstained with haematoxylin, and slides were imaged under a light microscope. Negative controls excluded the primary antibody.

### 3.6 Cell death assays

Cell death was assessed by LDH release and PI assays. For LDH release assays, the LDH released from cells in mouse BALF was detected by a cytotoxicity detection kit (Beyotime; Cat: C0016). For PI assays, MLE12 cell death cocultured with iBMDMs or neutrophils was evaluated by staining with PI (Sigma–Aldrich; Cat: No. P4863). Dead cells were identified under a fluorescence microscope based on PI uptake.

### 3.7 scRNA‐seq analysis

On day 3 and 7 post‐LPS‐induced lung injury, lineage tracing AT2 cells (CD45^−^CD31^−^EPCAM^+^tdTomato^+^) were sorted from PBS or FGF10‐treated ALI mice (*n* = 3 per group), with a viability rate >85%. Libraries were prepared using the Single Cell 3′ v 3 kit and sequenced with PE150 on an Illumina NovaSeq 6000. Quality control retained cells with >200 gene counts and <20% mitochondrial genes. Seurat (version 5.0.1) was employed for data normalisation, batch correction using Harmony and dimensionality reduction via UMAP.^36^ Subsequent to clustering with the Leiden algorithm, cell types were annotated utilising the CellMarker 2.0 database.^37^

### 3.8 Isolation and bulk RNA‐seq of epithelial cells

AECs from mice were isolated through a multi‐step process involving negative selection with CD31 (BioLegend; Cat#: 127411) and CD45 (BioLegend; Cat#: 03014) biotinylated antibodies, followed by positive selection using an EPCAM (BioLegend; Cat#: 118204) biotinylated antibody. This procedure was conducted utilising the MagCellect magnetic column system (R&D Systems). After sorting, total RNA was extracted and RNA quality was assessed; then RNA‐Seq was performed on the Illumina platform. DEGs were analysed using the limma package in R.^38^

### 3.9 Flow cytometry analysis

Mouse lung tissue was processed into single‐cell suspensions, preincubated with Mouse TruStain FcX™ antibodies (BioLegend; Cat: 101320) and treated with Fixable Viability Stain 780 (BD Bioscience; Cat: 406507) to exclude dead cells. Then, cells were stained with fluorescently labelled antibodies, including CD45 (BioLegend; Cat: 103112), CD31 (BioLegend; Cat: 102410, 102419), EPCAM (BioLegend; Cat: 118208, 118227), cleaved caspase‐3 (Cell Signaling Technology; Cat: 9664T), CD11b (BioLegend; Cat: 101215), Ly6G (BioLegend; Cat: 127627), F4/80 (BioLegend; Cat: 123109), FGFR2b (Cell Signaling Technology; Cat: 37374), Brilliant Violet 421™ Donkey anti‐rabbit IgG (BioLegend; Cat: 406410). After washing, cells were analysed using a BD LSRFortessa™ cytometer. Data were processed with FlowJo software. Gates were set using isotype and FMO controls.

### 3.10 Immunofluorescence

Lung tissue sections were fixed in 4% paraformaldehyde, permeabilised with.2% Triton X‐100 and blocked with 5% BSA. After overnight incubation with primary antibodies at 4°C, sections were stained with secondary antibodies for 1 h at room temperature. DAPI was used for nuclear counterstaining. Images were captured using a confocal microscope (Olympus FV3000), and fluorescence intensity was analysed with ImageJ. Negative controls were included by omitting primary antibodies. The antibodies used included EPCAM (BioLegend; Cat: 118211), cleaved caspase‐3 (Cell Signaling Technology, Cat: 9664T), cleaved caspase‐8 (Cell Signaling Technology; Cat: 8592T) and p‐RIPK1 (ThermoFisher; Cat: pa104645).

### 3.11 Cell culture and stimulation

Immortalised macrophage cell line iBMDMs (C57BL/6 mice‐derived) was provided by Professor Xing Liu (Institute Pasteur of Shanghai, China) and cultured in DMEM (Gibco) with 10% FBS (Gibco; Cat: 10099141c), 1×penicillin/streptomycin (Gibco; Cat: 15140122), 1×GlutaMAX (Cat: 15630‐080) and.04% 2‐mercaptoethanol. MLE12 cells (ATCC; CRL‐2110) were cultured in DMEM/F12 (Gibco) with 10% FBS and 1×penicillin/streptomycin. Both cell lines were authenticated by STR analysis and tested negative for mycoplasma. Neutrophils were isolated from mouse bone marrow by flushing femurs and tibias with pre‐cooled RPMI 1640 (Gibco). Mouse bone marrow cells were separated using Percoll gradient (80, 65 and 55%). Red blood cells were lysed. And the remaining cells were cultured in RPMI 1640 with 10% FBS and 1×penicillin/streptomycin. For evaluating the regulatory effects of FGF10, MLE12, iBMDMs and neutrophils were pretreated with FGF10 for 6 h. MLE12 cells were then co‐cultured with iBMDMs or neutrophils and stimulated with LPS (1 µg/mL), Compound C (5 µM) or AICAR (1 mM) for 6 h.

### 3.12 Immunoblotting

Cell lysates were prepared in RIPA buffer (Beyotime; Cat: P0013) with protease and phosphatase inhibitors. Protein concentrations were determined by the BCA assay. And equal amounts were separated by SDS‐PAGE and transferred to PVDF membranes. After blocking with 5% milk in TBST, membranes were incubated overnight with primary antibodies at 4°C, followed by HRP‐conjugated secondary antibodies for 1 h. Protein bands were visualised using ECL and imaged with a ChemiDoc system, with band intensities quantified using ImageJ software. The antibodies included β‐actin (Proteintech; Cat: 81115), tubulin (Proteintech; Cat: 11224‐1‐AP), p‐RIPK1 (Cell Signaling Technology; Cat: 53286), RIPK1 (Cell Signaling Technology; Cat: 3493), caspase 8 (Cell Signaling Technology; Cat: 4790), caspase 3 (Cell Signaling Technology, Cat: 9662), GSDME (Abcam, Cat: ab215191), FGF10 (R&D Systems, Cat: AF6224‐SP), FGFR2b (Abcam; Cat: ab10648), AMPK (Cell Signaling Technology; Cat: 2532) and p‐AMPK (Cell Signaling Technology; Cat: 2535s).

### 3.13 Real‐time quantitative PCR

Total RNA was extracted from lung tissue or cells using TRIzol reagent (Sigma). cDNA was synthesised using the FastKing RT Kit (Tiangen; Cat: KR116‐02) as per instructions. qRT‐PCR was performed with SYBR Green Master Mix (Yeasen; Cat: 11203ES08) on the RT‐PCR 384 system (Biored). The forward (F) and reverse (R) primer sequences for the target genes are listed in Table S1.

### 3.14 High‐performance liquid chromatography analysis of AMP, ADP and ATP

AMP, ADP and ATP levels were quantified by high‐performance liquid chromatography (HPLC) using a C18 reverse‐phase column (250 mm × 4.6 mm, 5 µm; Waters). Tissue or cell samples were homogenised in.6 M perchloric acid, centrifuged and neutralised with KOH. The HPLC system (Agilent 1200) was equipped with a UV detector (254 nm). The mobile phase consisted of 10 mM ammonium acetate buffer (pH 6.0) and acetonitrile, with a flow rate of 1 mL/min. Concentrations were determined by comparing sample peak areas to those of known standards. All assays were conducted in triplicate.

### 3.15 Statistical analysis

Data analyses were performed using GraphPad Prism 8.0 (La Jolla, California, USA), FlowJo 7.6.5 (Ashland, OR, USA). All quantitative data are presented as mean ± standard deviation (SD). The statistical significance of differences among treatment groups was determined by 2‐sided *t*‐test or one‐way ANOVA. The significance level was set at ^*^ *p* < .05, ^**^ *p* < .01 and ^***^ *p* < .001.

## 4 DISCUSSION

ARDS / ALI is a critical lung disease characterised by excessive inflammation, diffuse alveolar damage and severe hypoxemia.^39^ Despite progress in supportive care, like mechanical ventilation, effective treatments are scarce, highlighting the need for new therapeutic targets. Our study reveals a significant reduction in serum FGF10 levels in ARDS patients, which correlates with P/F ratio, hospitalisation days and mortality rates. In the animal experiments, we observed that FGF10 intervention significantly mitigated inflammatory responses and ALI in mice. Leveraging the analysis of scRNA‐seq of lineage tracing AECs and in vitro co‐culture experiments, we demonstrated that FGF10 prevents AEC pyroptosis induced by macrophages or neutrophils via inhibiting the activation of the AMPK–RIPK1/caspase‐8/caspase‐3/GSDME pathway. To our knowledge, this is the first study to clarify how FGF10 prevents AEC pyroptosis triggered by different immune cell infiltrations in different ways.

Programmed cell death contributes to diffuse alveolar damage.^40^ Correspondingly, our scRNA‐seq analysis demonstrated that FGF10 treatment reduced the expression of cell death‐related molecules in AECs – including *Xiap, Bax, Ripk1, Casp3, Casp8* and *Casp9*, which are involved in the regulation of both pyroptosis and apoptosis. Pyroptosis represents a pro‐inflammatory form of programmed cell death, leading to the release of pro‐inflammatory cytokines and cell debris.^6^ In contrast, apoptosis is often regarded as a ‘silent’ form of programmed cell death because it does not directly elicit an inflammatory response; instead, it facilitates the rapid removal of apoptotic bodies, thereby maintaining tissue homeostasis.^41^ Thus, we focused on pyroptosis in ALI.

In the in vitro model, it was observed that LPS alone did not induce pyroptosis in epithelial cells in the absence of immune cell co‐culture; however, under co‐culture conditions, LPS effectively triggered the pyroptotic pathway in epithelial cells. Our previous research indicated that TNF‐α, derived from macrophages, activates GSDME‐mediated pyroptosis in AECs.^24^ We supposed that LPS might induce pyroptosis of epithelial cells in vivo by increasing the source of TNF‐α and/or enhancing the downstream pathway elicited by TNF‐α. Our further results showed that FGF10 was unable to reduce TNF‐α release from macrophages, which are the primary source of TNF‐α.^42^ However, FGF10 inhibited the RIPK1/caspase‐8/caspase‐3/GSDME pathway triggered by TNF‐α within AECs. Taken together, we propose that during the macrophage‐infiltration‐dominated phase of ALI, FGF10 mitigates pyroptosis in AECs by preventing the downstream pathway.

Notably, in a co‐culture model of AECs with neutrophils, FGF10 not only inhibited the RIPK1/caspase‐8/caspase‐3/GSDME pathway but also lessened TNF‐α, IL‐6 and IL‐1β secretion from neutrophils in AECs. Of note, our findings suggest that FGF10 prevented caspase‐3/GSDME‐mediated pyroptosis in neutrophils (Figure S4). Previous research showed that lytic neutrophil releases TNF‐α via the PR3/caspase‐3/GSDME pathway.^28^ We propose that FGF10 may prevent pyroptosis in AECs by stopping neutrophils from undergoing another pyroptotic pathway, the caspase‐3/GSDME pathway and releasing TNF‐α. Thus, during the neutrophil‐infiltration‐dominated ALI phase, FGF10 can inhibit neutrophil pyroptosis to reduce TNF‐α release, as well as prevent AEC pyroptosis via FGFR2b. However, it is important to note that FGF10 did not affect pyroptosis in macrophages. In the ALI mice model, we observed a reduction in TNF‐α, IL‐6 and IL‐1β levels in BALF after FGF10 treatment. This reduction may be explained that neutrophil infiltration dominates in the acute phase of ALI.^43^

LPS‐induced ALI has the potential to induce oxidative stress in AECs.^44^ Under oxidative stress or energy‐deficient conditions, the activation of AMPK maintains cellular energy balance by promoting ATP synthesis and inhibiting ROS‐generating pathways.^45^ A recent study revealed that FGF10 could significantly reduce ROS levels in adipocytes, thus mitigating cellular injury.^13^ Our study found that in ALI, FGF10 boosts ATP synthesis and inhibits AMPK phosphorylation. Furthermore, a previous study has reported that AMPK phosphorylation promotes RIPK1 phosphorylation at Ser415, thereby suppressing RIPK1 activity, which is involved in cell survival, apoptosis, necroptosis, pyroptosis and inflammatory signalling.^46^, ^47^ In addition, AMPK inhibition has been shown to block pyroptosis during Yersinia infection.^33^, ^48^ Phosphorylation of RIPK1 at Ser166 induces caspase‐8 cleavage, driving pyroptosis.^49^ Similarly, we found that phosphorylation of AMPK in AECs can induce phosphorylation of RIPK1 at Ser^166^. Moreover, our findings demonstrated that FGF10 suppresses RIPK1/caspase‐8/caspase‐3/GSDME pathway in AECs, indicating that FGF10 prevents AEC pyroptosis by modulating the AMPK–RIPK1 interaction in ALI. Ulteriorly, we found that inhibiting AMPK reduced RIPK1 phosphorylation and blocked caspase‐8/caspase‐3/GSMDE‐mediated pyroptosis, whereas activation of AMPK had an opposite effect. This effect was rescued by FGF10. We also observed a reduction in total AMPK protein, which may reflect FGF10‐induced downregulation of AMPK expression or enhanced protein degradation and will be examined in future studies. Taken together, we concluded that FGF10 restored ATP levels to attenuate RIPK1 phosphorylation via AMPK, thereby disrupting pyroptosis in the AECs.

FGF10 is well recognised for its critical role in embryonic development and lung stem cells regeneration.^50^, ^51^, ^52^ It is also known to modulate the Wnt signalling pathway, which plays a pivotal role in lung epithelial repair and regeneration.^53^ However, the clinical significance of FGF10 in ARDS remains unclear. In this study, we observed significantly decreased serum levels of FGF10 in ARDS patients, which correlate with P/F ratio, hospitalisation days and mortality rates. Although these clinical data do not establish causality, they highlight FGF10 as a potential prognostic biomarker in ARDS. A previous randomised controlled trial has shown that keratinocyte growth factor can reduce inflammation by upregulating the anti‐inflammatory cytokines.^54^ FGF family members have also been reported to promote epithelial repair and attenuate lung injury by modulating inflammation.^54^, ^55^ Here, we further demonstrated that FGF10 not only attenuates inflammation but also uniquely inhibits pyroptosis via the AMPK–RIPK1–caspase‐8–caspase‐3–GSDME pathway. This finding suggests that FGF10 not only participates in epithelial regeneration but also modulates inflammation, representing a novel mechanistic dimension within the FGF family's role in lung injury. Therefore, we propose that FGF10 mitigates lung injury by inhibiting AEC apoptosis induced by different immune cell infiltrations through distinct mechanisms. These properties position FGF10 as a potential therapeutic agent for ARDS.

The study has several limitations. First, while our in vitro experiments demonstrated the role of FGF10 in inhibiting AEC pyroptosis, these findings have not been validated in an in vivo ALI mice model with macrophages/neutrophils depletion. Consequently, our in vitro results are insufficient to understand the mechanisms by which FGF10 operates within a complex immune environment. Second, we observed a correlation between serum FGF10 levels and ARDS patient prognosis, but the small sample size (*n* = 28) limits the generalisability of this finding. Large‐scale studies are warranted to confirm these observations, while longitudinal patient sampling and targeted loss‐/gain‐of‐function animal studies are required to determine directionality. Third, although our study primarily focuses on GSDME‐mediated pyroptosis, other programmed cell death pathways – including apoptosis, necroptosis and ferroptosis – may coexist or interact with pyroptotic signalling under inflammatory conditions.^56^, ^57^Future studies should further clarify how FGF10 influences the balance and crosstalk among these pathways to maintain alveolar homeostasis. Furthermore, our exploration of FGF10's therapeutic potential is based on preclinical data. We plan to conduct a randomised controlled trial to evaluate the efficacy and safety of FGF10 in ARDS treatment. Thus, further research is essential to fully elucidate the role and therapeutic potential of FGF10 in this context.

In conclusion, our study reveals a marked decrease of serum FGF10 levels in ARDS patients, correlating with P/F ratio, hospitalisation days and mortality rates. Our research demonstrates that FGF10 treatment alleviates ALI in vitro and in vivo by inhibiting the AMPK–RIPK1/caspase‐8/caspase‐3/GSDME pathway‐mediated pyroptosis. Furthermore, FGF10 appears to exert effects across various inflammatory phases characterised by the predominance of either macrophages or neutrophils. Our work positions FGF10 as a promising therapeutic candidate for ARDS/ALI, offering a precision‐targeted approach to disrupt the inflammatory cascade.

## AUTHOR CONTRIBUTIONS

Tianchang Wei, Xiaoyan Chen and Cuiping Zhang designed the research, interpreted the data and wrote the manuscript. Tianchang Wei, Jian Xu and Weiqi Mao analysed bulk RNA‐seq data and carried out experiments and wrote the manuscript. Tianchang Wei, Xiaoyan Chen and Jian Xu shared the co‐first authorship. Yuhan Wang and Yufan Li helped to develop the experiments. Wenting Jin and Cuicui Chen helped to collect human samples. Yuanlin Song, Xiaoyan Chen and Cuiping Zhang supervised the study, provided scientific insight and reviewed and edited the manuscript.

## CONFLICT OF INTEREST STATEMENT

The authors declare no conflicts of interest.

## ETHICS STATEMENT

The protocols were approved by the Committees on Animal Research of Fudan University (202408FD0001) and the Clinical Research Ethics Committee of Zhongshan Hospital of Fudan University (Approval Number: B2021‐128 and B2021‐183R).

## CONSENT

Written informed consent for publication was obtained from all participants.

## Supporting information



Supporting Information

## Data Availability

Data available upon request from the authors.
