# Supplementary material for: Roll with the punches: Fibroblast growth factor 10 alleviates pyroptosis of alveolar epithelial cells in different immune niches
Source: Clin Transl Med. 2026 Jan 2;16(1):e70569. doi: 10.1002/ctm2.70569 (PMC12759039; doi:10.1002/ctm2.70569)
Supplement: Supplementary file 1 — Supporting Information [file CTM2-16-e70569-s001.pdf]

Supplementary data

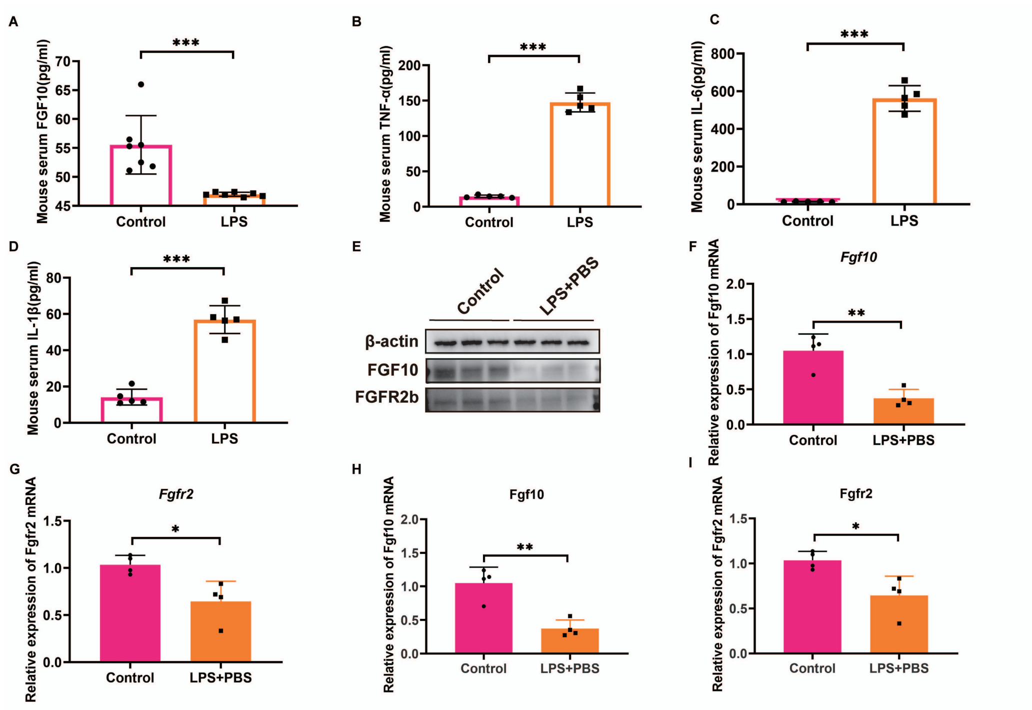

Figure S1. Serum FGF10 is decreased in LPS-induced ALI mice.

(A) Serum FGF10 levels measured by ELISA in LPS-treated mice versus healthy controls. (B-D) Elevated serum pro-inflammatory cytokines in LPS-treated mice and healthy controls: TNF-α (B), IL-6 (C), and IL-1β (D). (E) Western blot of lung tissue lysates probed for FGF10 and FGFR2b. (F-G) Statistical analysis of FGF10 (F) and FGFR2b (G) protein expression normalized to β-actin. (H-I) qRT-PCR analysis of *Fgf10* (H) and *Fgfr2b* (I) mRNA levels in lung tissues. Statistical significance was determined by a two-sided *t*-test. At least three independent experiments are carried out. Data expressed as mean ± SD (n=3-5).

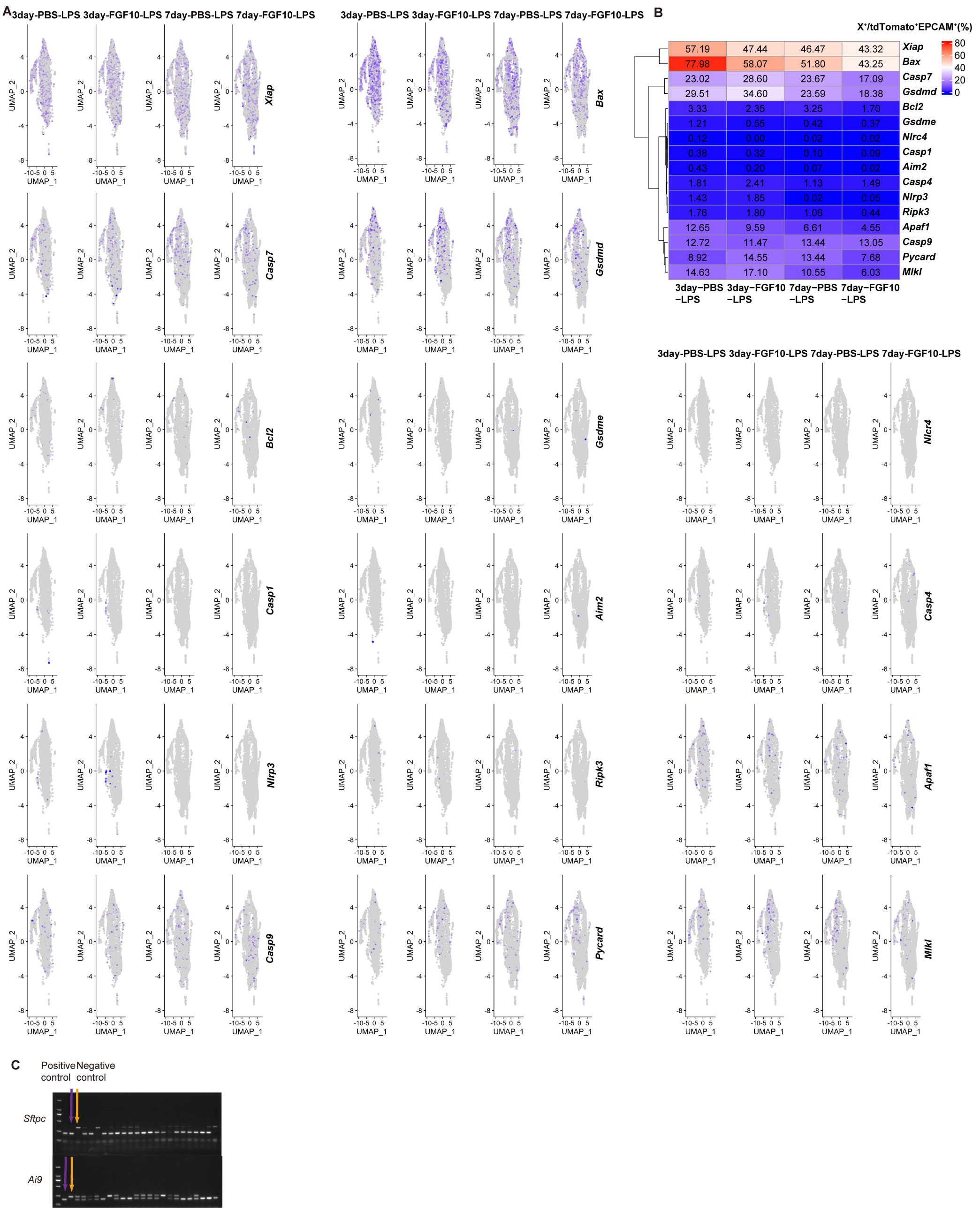

Figure S2. Single-cell profiling of cell death-related molecules in alveolar epithelial cells.

(A) UMAP projection of scRNA-seq data from LPS-treated alveolar epithelial cells (td Tomato^{+}EPCAM^{+}), colored by expression levels of cell death-associated molecules. (B) Heatmap depicting the proportion of expressing death-related molecules (rows) across experimental groups (columns). Data was processed using Seurat v4.0 (clustering resolution=0.8).

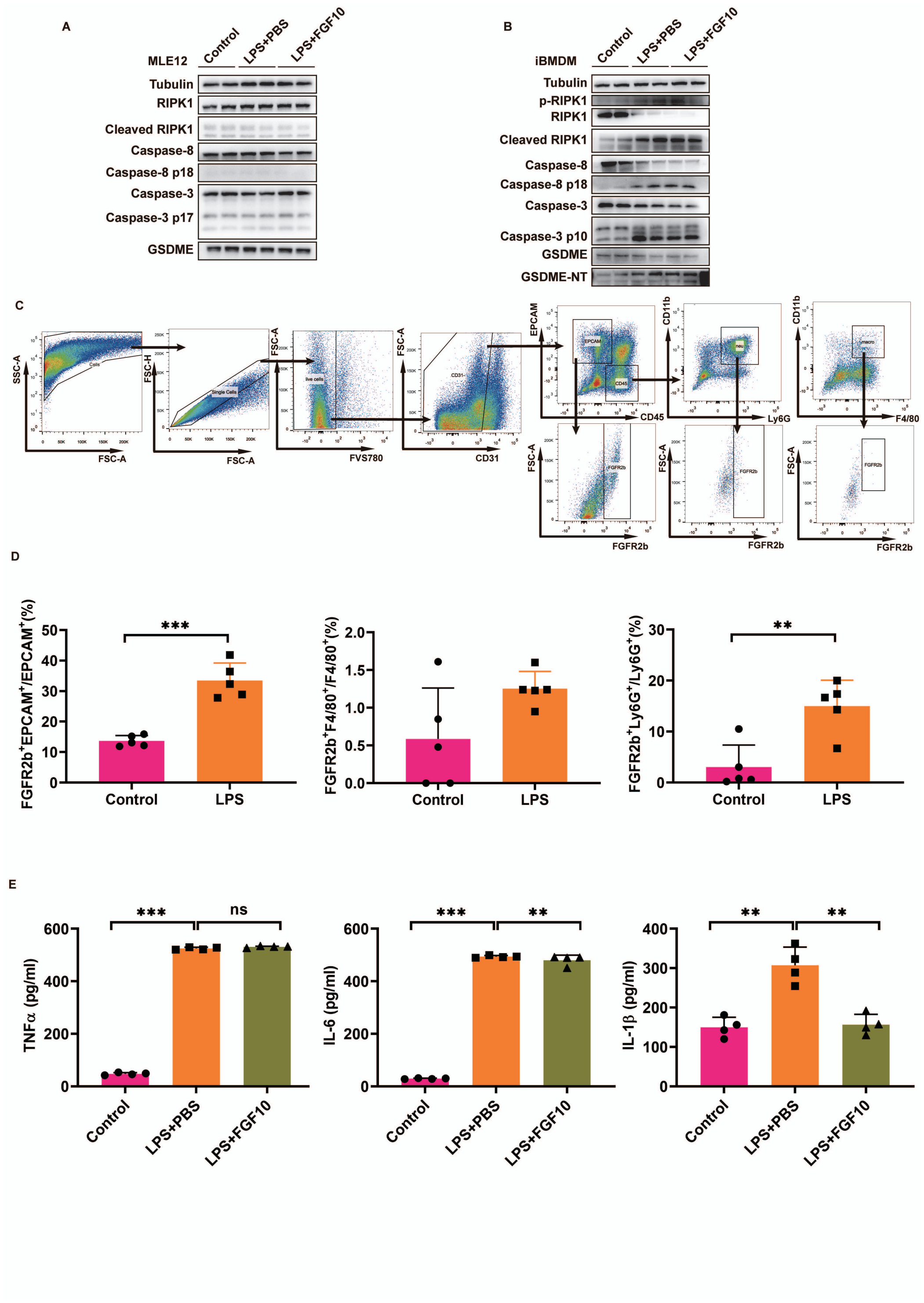

Figure S3. Validation of pyroptosis in MLE12 cells or iBMDMs and FGFR2b expression profiles. (A) WB analysis of RIPK1, caspase-8, caspase-3, and GSDME in LPS-treated MLE12 cells (6 h, 1 μg/mL LPS) alone. (B) WB analysis in LPS-stimulated iBMDM cells (24 h, 1 μg/mL LPS). (C) Flow cytometry gating strategy for FGFR2b⁺ cells. (D) FGFR2b expression across AECs,

macrophages, and neutrophils in control vs LPS-treated mice. (E) TNF-α, IL-6, and IL-1β in MLE12-iBMDM co-culture supernatants detected by ELISA. Statistical significance was determined by one-way ANOVA with Tukey's post hoc test. At least four independent experiments are carried out. Data are shown as mean ± SD (n=4-5, *p<0.05, **p<0.01, ***p<0.001).

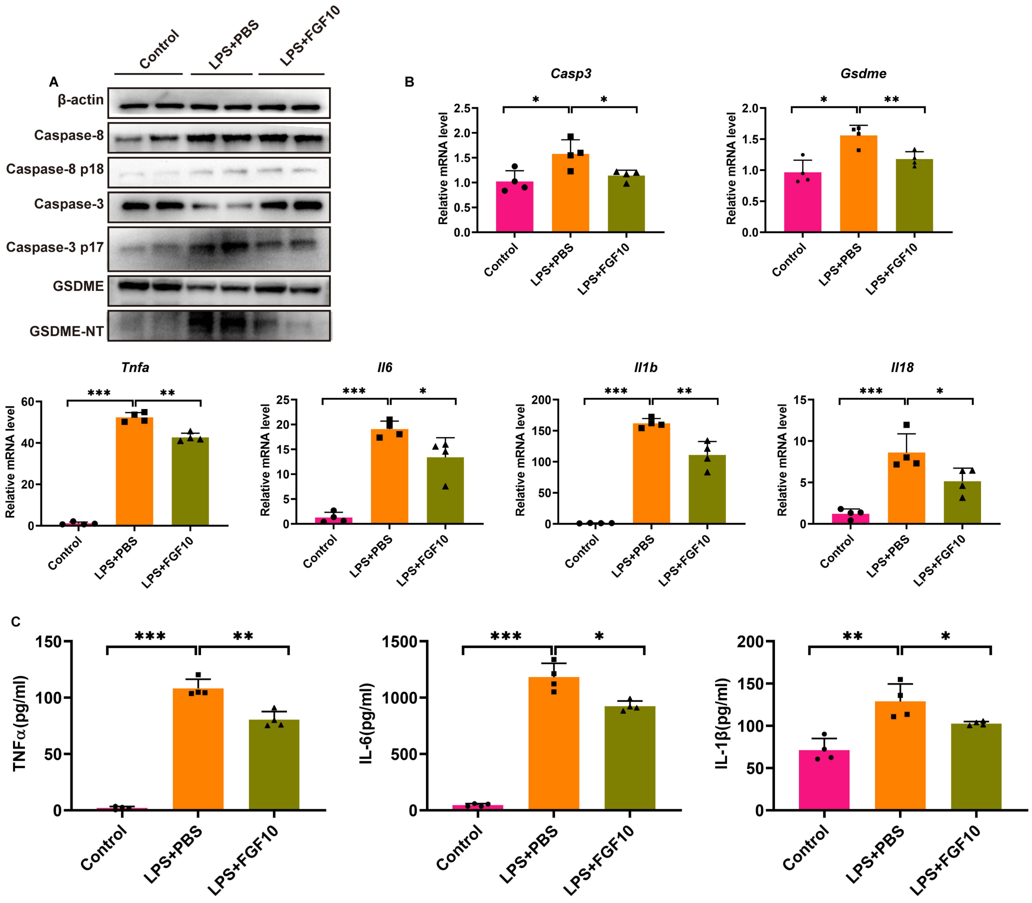

Figure S4. FGF10 suppresses neutrophil pyroptosis and cytokine secretion in LPS-stimulated co-culture systems.

(A) Western blot analysis of caspase-8, caspase-3, GSDME in LPS-treated neutrophils (1 μg/mL, 24 h) with or without FGF10 (100 ng/mL). (B) qRT-PCR analysis of *Casp3, Gsdme*, and pro-inflammatory cytokines (*Tnfa, Il6, Il1b, Il18*) in neutrophils. (C) ELISA detection of TNF-α, IL-6,

and IL-1β in MLE12-neutrophil co-culture supernatants. Statistical significance was determined by one-way ANOVA with Tukey's post hoc test. Four independent experiments are carried out. Data are shown as mean ± SD (n=4, *p<0.05, **p<0.01, ***p<0.001).

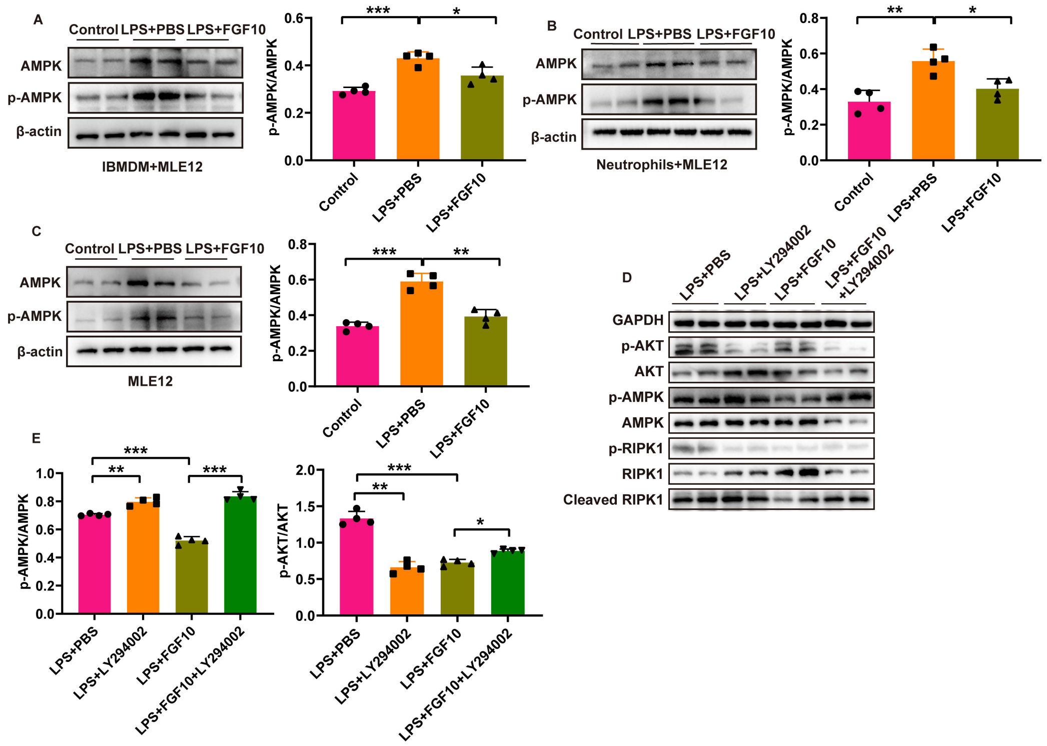

Figure S5. FGF10 induces AMPK activation across mono- and co-culture MLE12.

(A) WB and densitometric analysis of phospho-AMPKα (Ser166) and total AMPK in MLE12 cells co-cultured with iBMDMs. (B) Parallel analysis in MLE12-neutrophil co-cultures. (C) Parallel analysis in LPS-treated MLE12 alone. (D-E) WB and densitometric analysis in MLE12 cells co-cultured with iBMDMs. Statistical significance was determined by one-way ANOVA with Tukey's post hoc test. Four independent experiments are carried out. Data are shown as mean ± SD (n=4, *p<0.05, **p<0.01, ***p<0.001).

WB merge

Primers in this article

Primers(Mus)	5'to3'
Gapdh-F	AGGTCGGTGTGAACGGATTTG
Gapdh-R	TGTAGACCATGTAGTTGAGGTCA
Fgf10-F	TTTGGTGTCTTCGTTCCCTGT
Fgf10-R	TAGCTCCGCACATGCCTTC
Fgfr2b-F	AATCTCCCAACCAGAAGCGTA
Fgfr2b-R	CTCCCCAATAAGCACTGTCCT
Gsdme-F	TGCAACTTCTAAGTCTGGTGACC
Gsdme-R	CTCCACAACCACTGGACTGAG
Casp3-F	ATGGAGAACAACAAAACCTCAGT
Casp3-R	TTGCTCCCATGTATGGTCTTTAC
Casp8-F	TGCTTGGACTACATCCCACAC
Casp8-R	GTTGCAGTCTAGGAAGTTGACC
Tnfa-F	CCCTCACACTCAGATCATCTTCT
Tnfa-R	GCTACGACGTGGGCTACAG
Il1b-F	GACCTTCCAGGATGAGGACA
Il1b-R	AGCTCATATGGGTCCGACAG
Il6-F	TAGTCCTTCCTACCCCAATTTCC
Il6-R	TTGGTCCTTAGCCACTCCTTC
Rlipk1-F	GAAGACAGACCTAGACAGCGG
Ripk1-R	CCAGTAGCTTCACCACTCGAC
Il18-F	GTGAACCCCAGACCAGACTG
Il18-R	CCTGGAACACGTTTCTGAAAGA

Figure 4

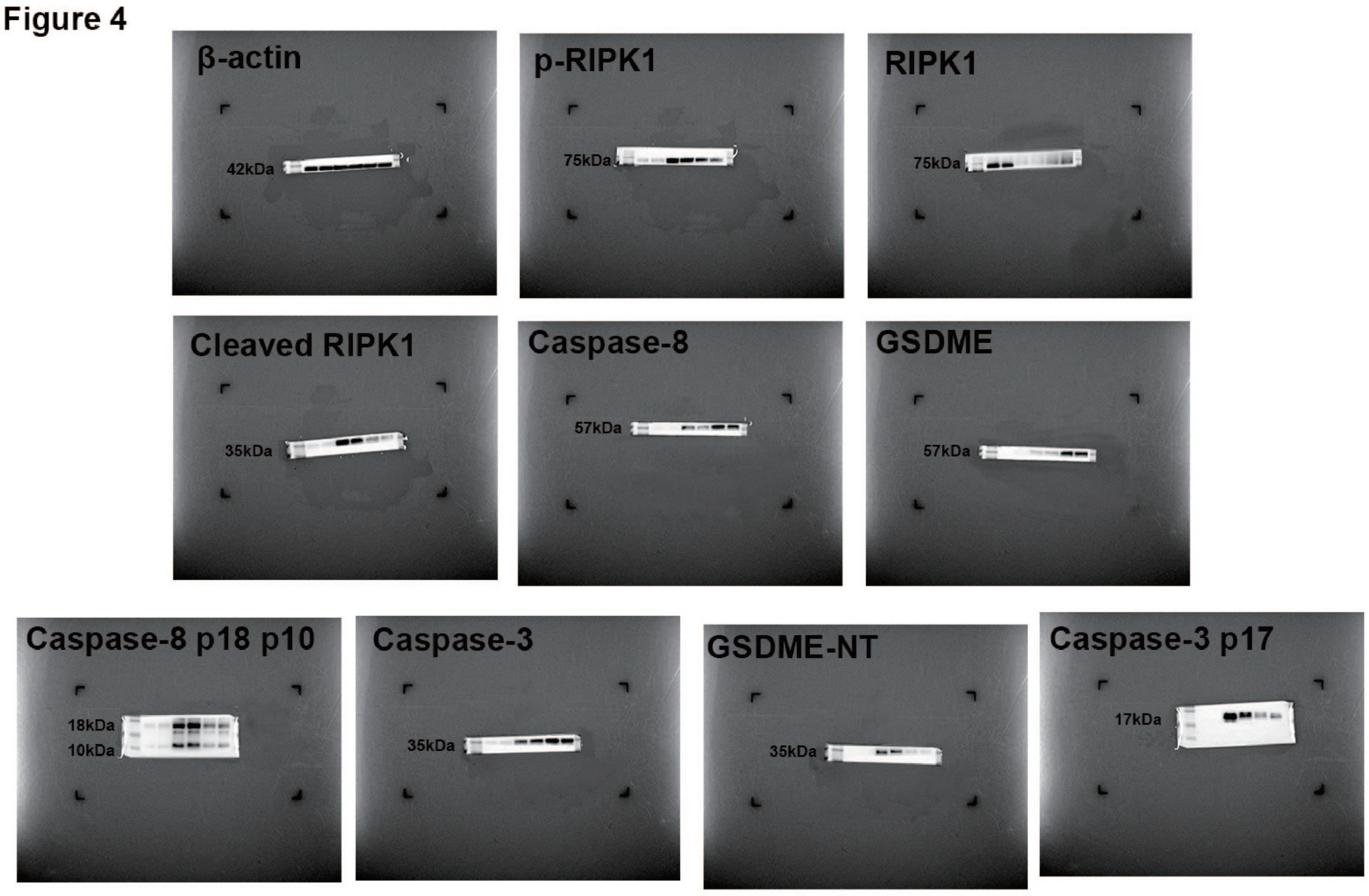

Figure 5F

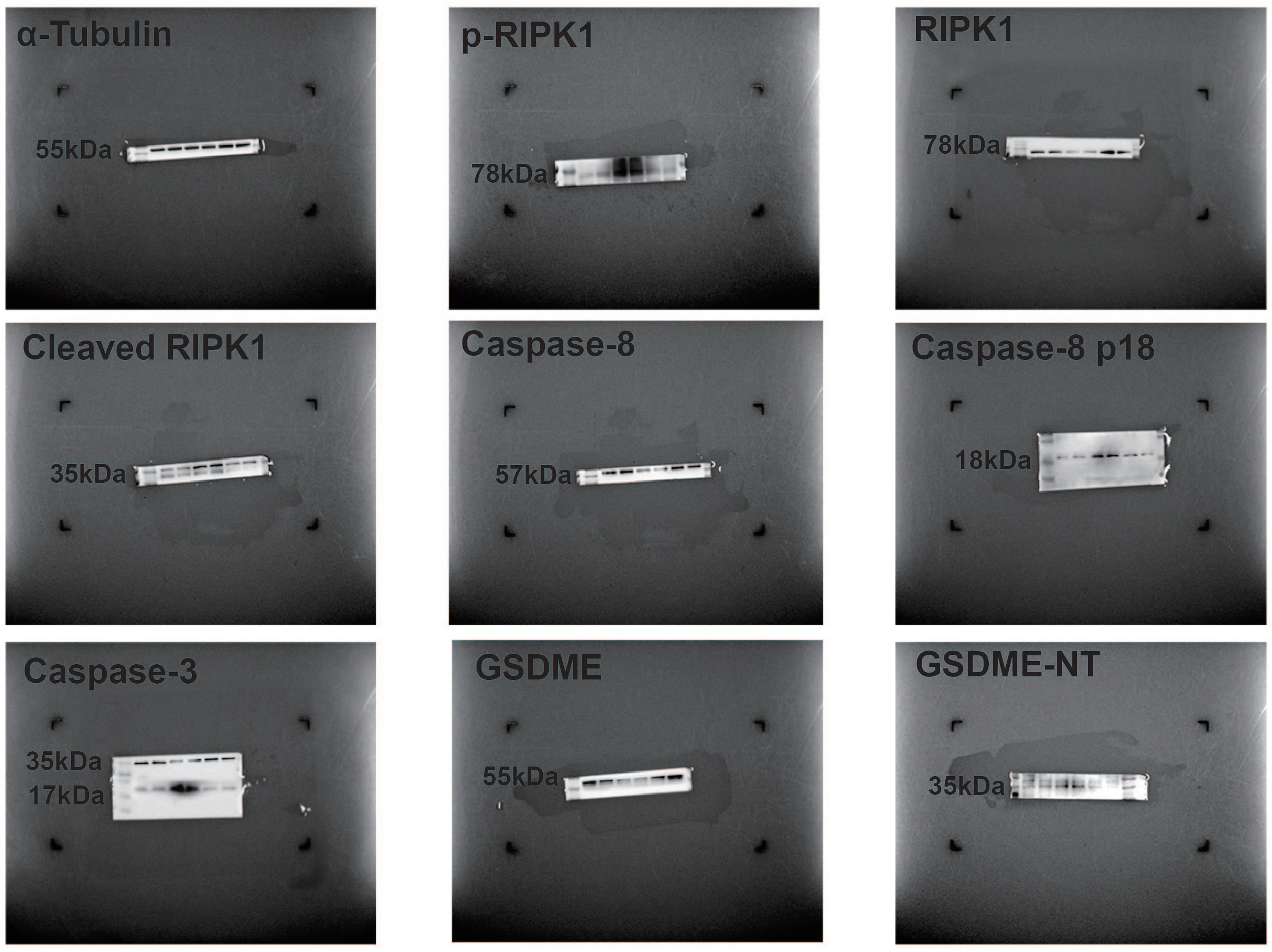

Figure 6D

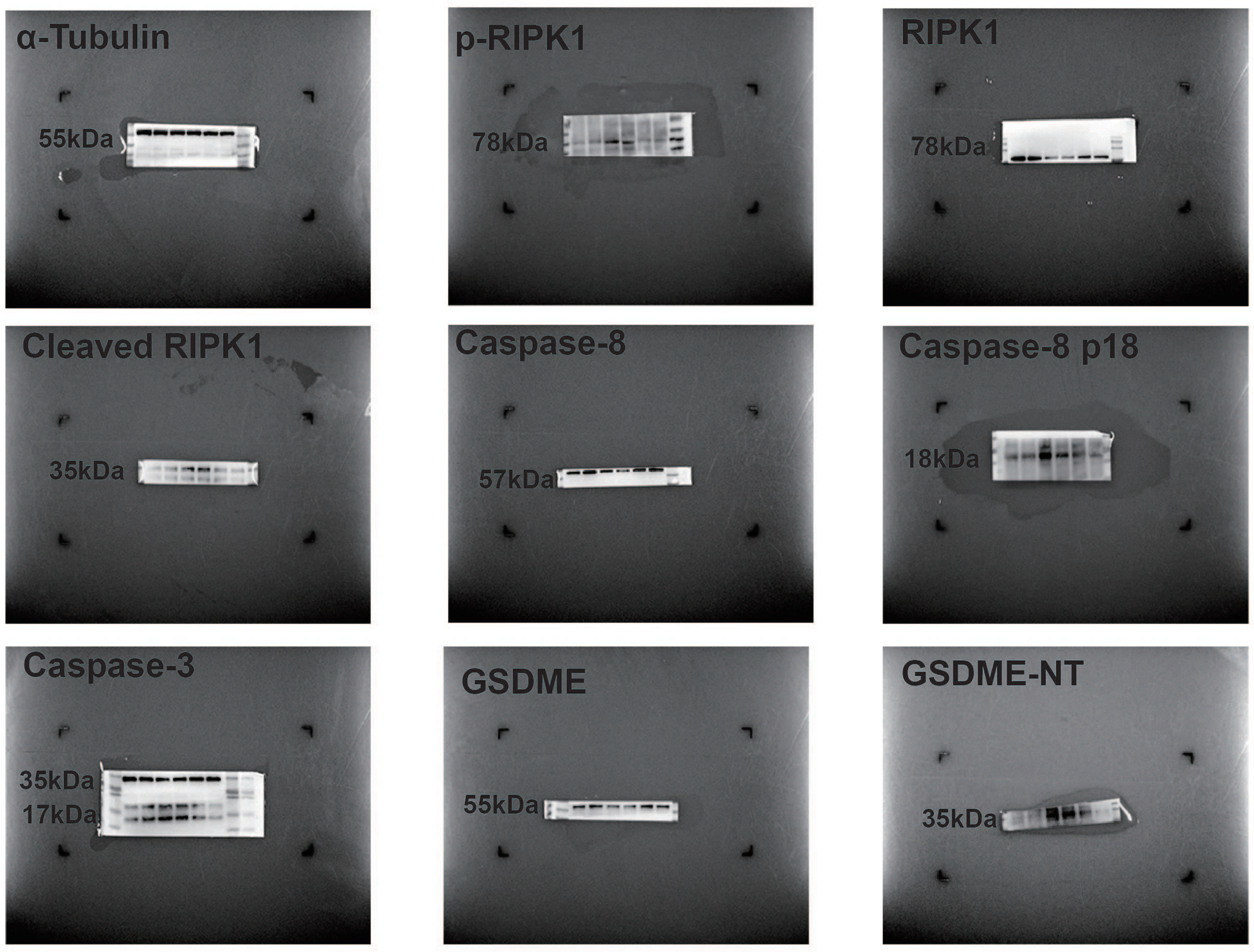

Figure 7D

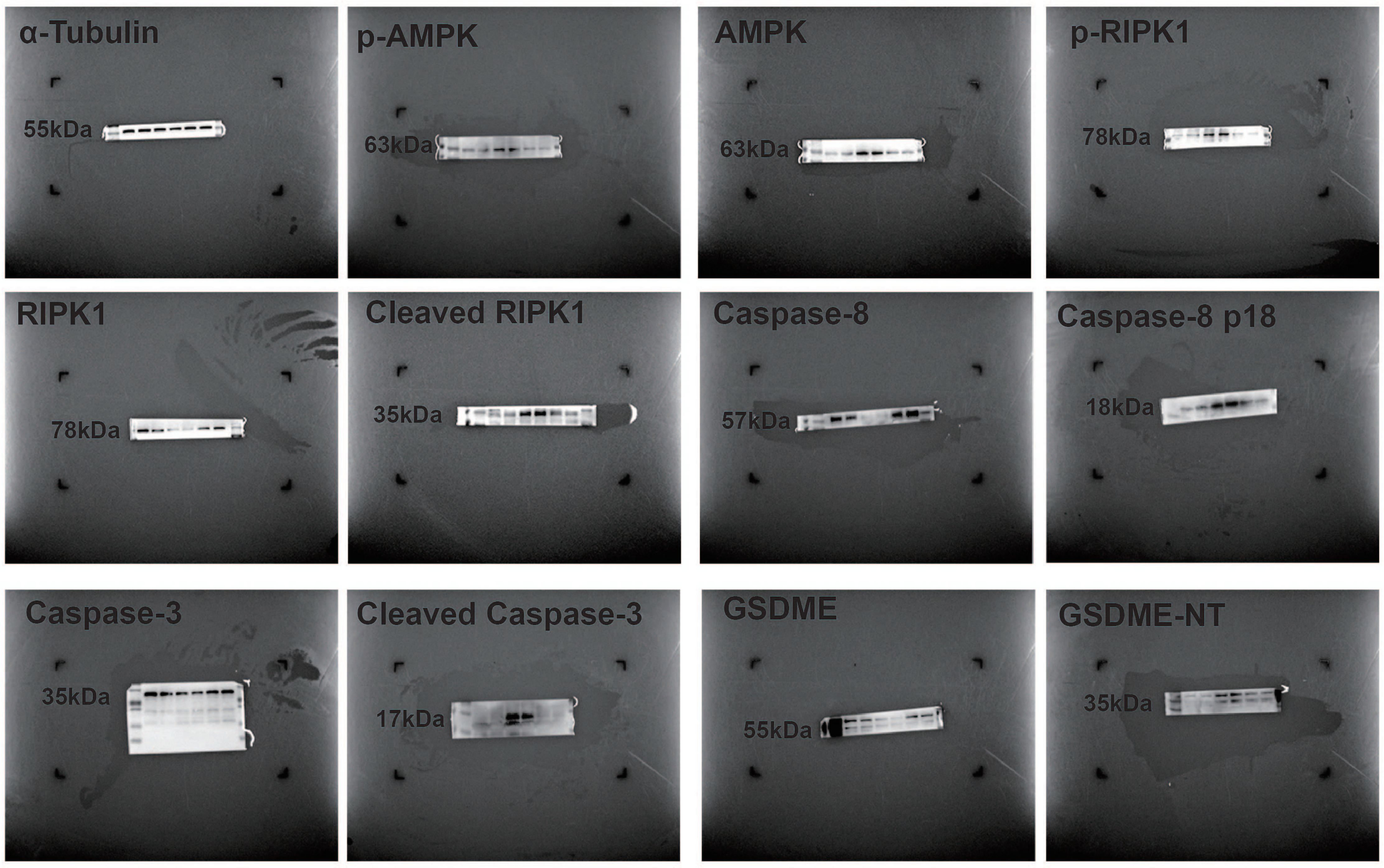

Figure 7F

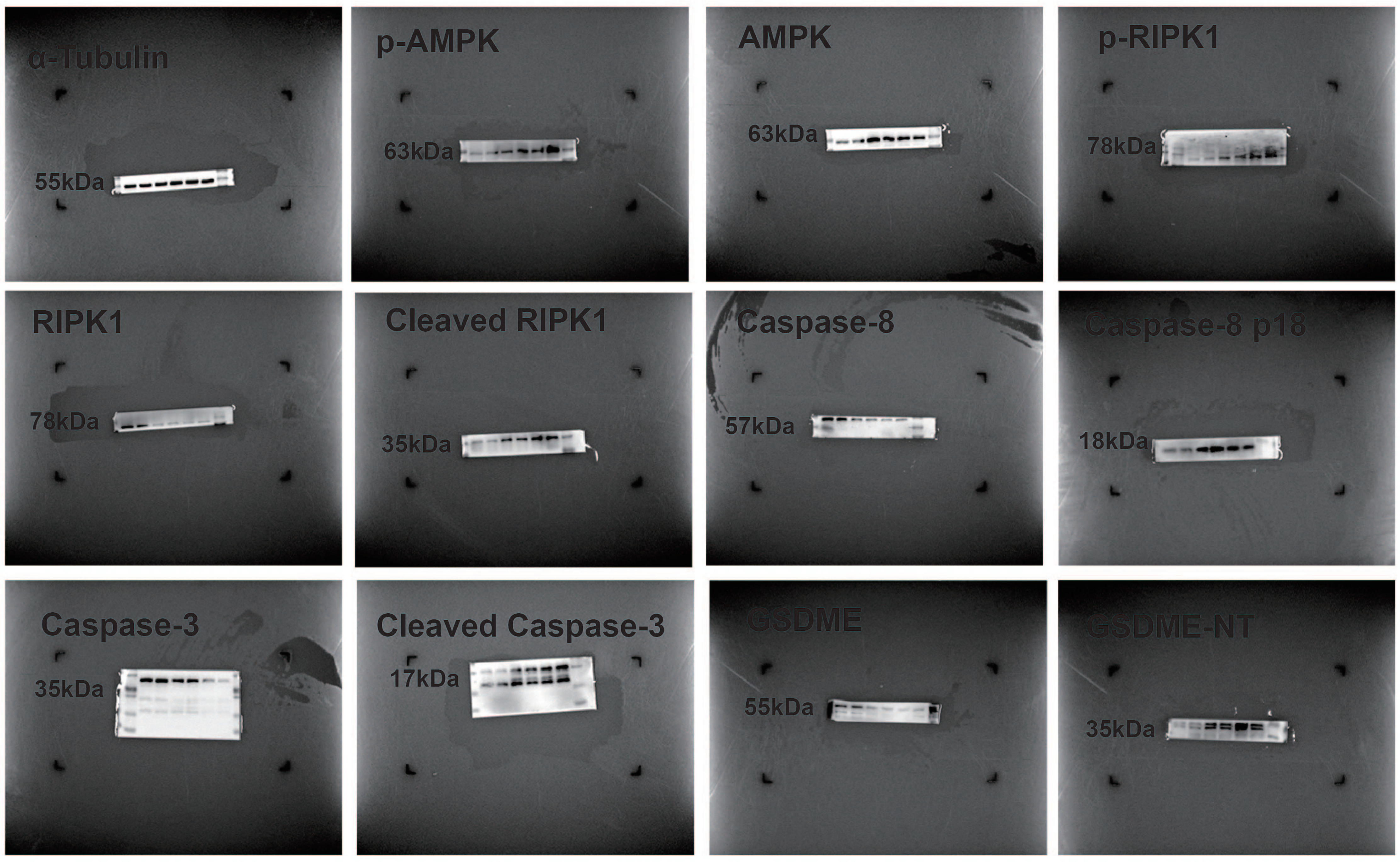

Figure 7H

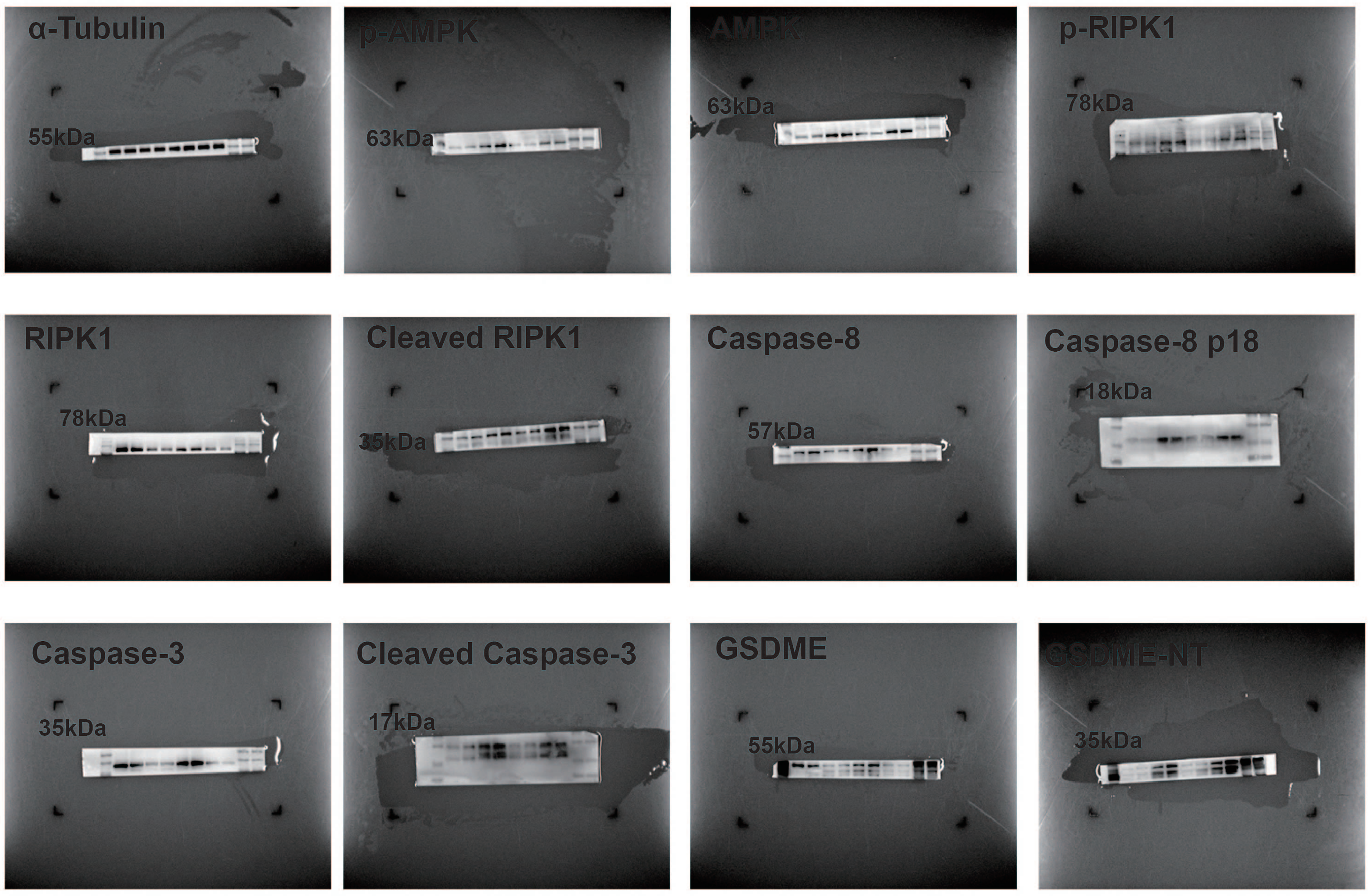

Figure S1

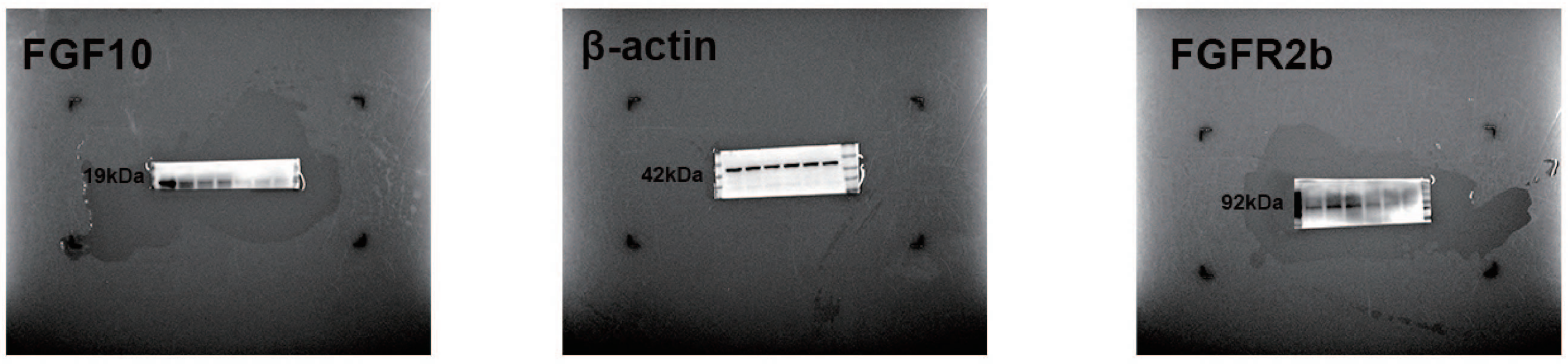

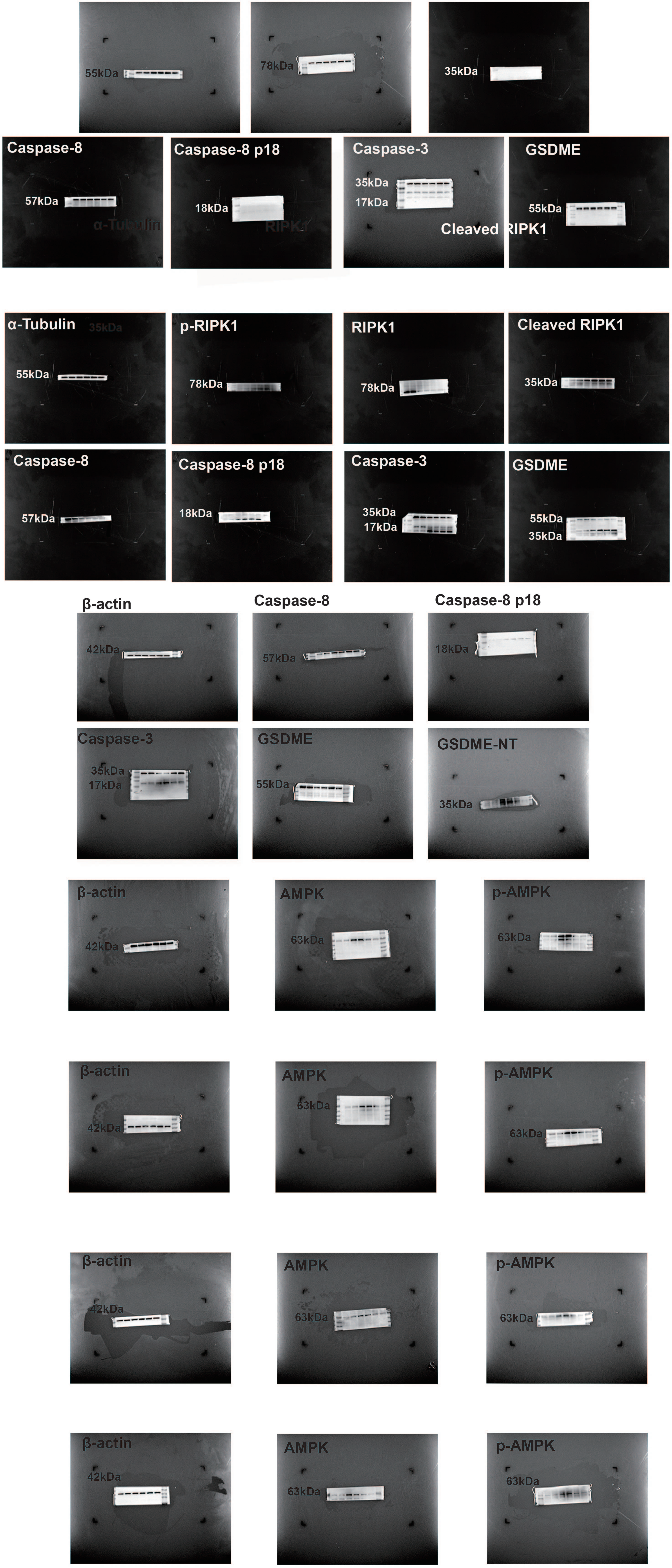
55kDa
78kDa
35kDa
Caspase-8
57kDa
Caspase-8 p18
18kDa
Caspase-3
35kDa
17kDa
Cleaved RIPK1
GSDME
55kDa
α-Tubulin
55kDa
p-RIPK1
78kDa
RIPK1
78kDa
Cleaved RIPK1
35kDa
Caspase-8
57kDa
Caspase-8 p18
18kDa
Caspase-3
35kDa
17kDa
GSDME
55kDa
35kDa
β-actin
42kDa
Caspase-8
57kDa
Caspase-8 p18
18kDa
Caspase-3
35kDa
17kDa
GSDME
55kDa
GSDME-NT
35kDa
β-actin
42kDa
AMPK
63kDa
p-AMPK
63kDa
β-actin
42kDa
AMPK
63kDa
p-AMPK
63kDa
β-actin
42kDa
AMPK
63kDa
p-AMPK
63kDa
β-actin
42kDa
AMPK
63kDa
p-AMPK
63kDa
